# Game Addiction Scale for Adolescents—Psychometric Analyses of Gaming Behavior, Gender Differences and ADHD

**DOI:** 10.3389/fpsyt.2022.791254

**Published:** 2022-03-09

**Authors:** Frida André, Ingrid Munck, Anders Håkansson, Emma Claesdotter-Knutsson

**Affiliations:** ^1^Department of Clinical Sciences Lund, Faculty of Medicine, Lund University, Lund, Sweden; ^2^Department of Education and Special Education, University of Gothenburg, Gothenburg, Sweden; ^3^Department of Clinical Sciences Lund, Psychiatry, Faculty of Medicine, Lund University, Lund, Sweden; ^4^Gambling Disorder Unit, Malmö Addiction Centre, Malmö, Sweden; ^5^Child and Adolescent Psychiatry, Regional Out-Patient Care, Lund University Hospital, Region Skåne, Lund, Sweden

**Keywords:** internet gaming disorder GASA, core approach, gender differences, psycho-social model, aligned factor scores

## Abstract

**Background:**

Internet gaming disorder (IGD) was recently added in the Diagnostic and Statistical Manual of Mental Disorder as a “condition for further studies.” There is no consensus regarding which rating scales should be used but many scholars suggest the GASA (Game Addiction Scale for Adolescents) and a ranking of the criteria, “the core approach” to avoid over-diagnosing of disordered gaming. Male gender and ADHD are commonly listed as risk factors for disordered gaming but little is known about sex differences in gaming and gender specific health correlates.

**Purpose:**

The present study aims to evaluate the core approach and the specific indicators of gaming behavior in GASA from a multifactorial perspective and explore the gender differences in a clinical setting, focusing on ADHD.

**Patients and Methods:**

Children and adolescents aged 8–18 years (*n* = 144) from Child and adolescent psychiatry (CAP) in Skane were assessed with the GASA. Psychometric analyses including confirmatory factor analyses (CFA) and structural equation modeling (SEM) were used to identify well-defined constructs and gender differences. Refined factor scores for single constructs were the outcome of alignment, a procedure for assessing measurement equivalence across gender. New model-based gaming behavior variables were used for descriptive statistics and ANOVA testing of gender differences.

**Results:**

The results confirm that the core approach two-factor model is valid for the CAP sample, as well as a theory based psycho-social model for gaming behavior with over consumption and negative social and emotional consequences. Our findings suggest that negative consequences of over consumption take a social direction for boys and an emotional direction for girls. Also, ADHD was significantly associated with over consumption of video games and the negative consequences thereof for girls.

**Conclusion:**

Guided by psychometric analyses, the GASA could be strengthened by advancing the questionnaire design and by adding complementary items in order to illuminate the complexity of gaming behavior. Our findings suggest that additional research on potential gender related discrepancies of disordered gaming is needed.

## Introduction

In the Diagnostic and Statistical Manual of Mental Disorders (DSM-5) the American Psychiatry Association (APA) identified Internet Gaming Disorder as a tentative diagnose; a “condition for further studies” (1). Nine criteria for IGD has been proposed: preoccupation, preoccupation with gaming; withdrawal, experience of unpleasant symptoms when gaming is taken away; tolerance, the need to spend increasing amounts of time engaged in games; loss of control, unsuccessful attempts to control participation in games; Give up other activities, loss of interest in previous hobbies and entertainment as a result of, and with the exception of, internet games; continuation, continued excessive gaming despite knowledge of psychosocial problems; deception, deceiving family members, therapists, or others regarding the quantity of gaming; escape, the use of games to avoid or relieve negative moods; and negative consequences, risking or losing an important relationship, job or education or career opportunity due to participation in games. Five of the nine criteria must be met within a year to be diagnosed as IGD (1). However, APA indicated that further clinical experience and research was needed before inclusion of IGD as a formal disorder (1).

In 2018, the World Health Organization (WHO) included Gaming Disorder (GD) in the 11th revision of the International Classification of Diseases (ICD 11) (2). According to ICD-11 a patient must exhibit three symptoms (impaired control, increasing priority given to gaming, continuation or escalation of gaming despite the occurrence of negative consequences) to be officially diagnosed with GD (2). Consequently, the criteria withdrawal and tolerance which concerns rather biological consequences is excluded from the ICD-11 GD diagnosis criteria.

There is no consensus regarding which rating scales should be used for diagnosing disordered gaming and different scales are used both in research and in clinical practice. Most studies have used the criteria for pathological gambling to define the pathological gaming (3, 4). Different researchers have used different cutoffs of the criteria to establish a diagnosis (5–8), others have focused strictly on online games (9), and some researchers have adopted their own criteria for disordered gaming (10, 11). One of the most frequently used questionnaires for disordered gaming in adolescents is the GASA (Game Addiction Scale for Adolescents) (12–16). GASA was developed specifically for adolescents. The items in the GASA relate to homework and relationship to parents, designed to correspond to the developmental stage of an adolescent (15). The adult version of the GASA; Game Addiction Scale (GAS) has been showed to provide both good reliability and validity and in a review of different instruments assessing disordered gaming King et al., found that GAS was one of two scales that provided the best clinical information for the diagnosis of disordered gaming (16). King et al. reviewed 32 different scales and found that GAS was one out of five tools that had greater evidential support regarding psychometric properties (16). Finserås et al. verified this finding in their evaluation of the adolescent version of the scale (GASA) in relationship to the nine criteria for disordered gaming suggested by the APA (17). In 2019 Donati et al. developed and evaluated a Video-Gaming-Scale—For Children (VGS-C), aiming to assess pathological gaming behavior in children specifically (18). The GASA has the advantage of being a well-established and well-proven assessment of disordered gaming (12–17). However, the scale has to our knowledge not yet been evaluated in a child and adolescent sample.

GASA was theoretically based on seven of the DSM-5 criteria for pathological gambling: salience (exaggerated preoccupation in thoughts and habits), tolerance, mood modification, withdrawal, relapse, conflicts, and problems (15). When diagnosing pathological gamblers, the DSM-5 requires at least half of their criteria to be met while scholars in the gaming research field prefer a ranking of the criteria, which they call “the core approach” (1, 5, 12–14, 19). These scholars believe that the criteria for tolerance, mood modification and cognitive salience are associated with engagement and not necessarily with addiction while the contrary is true for the criteria for withdrawal, relapse, conflicts and problems (5, 13, 14). The core approach thus distinguishes engaged gamers from problem- and addicted gamers by emphasizing the “core criteria” namely, withdrawal, relapse, conflict and problems in order to yield a more precise and relevant estimate of prevalence whereby a diagnosis of game addiction should be related to comorbidity and interference rather than high engagement (13, 14, 17, 20).

The psychometric properties of GAS have been tested among adult men in Switzerland showing satisfactory internal consistency (21), and in a population of Iranian adolescents supporting the measurement invariance also across gender (22). Brunborg et al. evaluated the core approach using a confirmatory factor analysis showing that a two-factor structure (peripheral criteria separated from core criteria) fitted their data better than the original one-factor structure. The same applied for groups of men and women, both aged 16–33 years and for those aged 34–74 years (13). However, when Brunborg et al. evaluated the two-factor solution no evidence was found for metric invariance, implicating that comparison between different subpopulations should be done with caution (13). Charlton and Danford contributed with an influential distinction between peripheral and core symptomatology in terms of gaming, early in the field of gaming research. Consistent with the Brunborg et al. research they considered cognitive salience, tolerance, and mood modification as a peripheral group of symptoms, though with a potential to develop into disordered gaming in certain circumstances (5, 19). Concordantly, they suggested an existence of a developmental process whereby the peripheral criteria precede the core criteria (5).

Jonsson et al. evaluated a self-test, GamTest, for online gambling, largely similar to GASA. These researchers identified two main components of early signs of problematic gambling: over consumption (OC) and negative consequences (NC) (23). The peripheral criteria correspond to over consumption and the core criteria to the negative consequences. The negative consequences items where further divided conceptually into a social and an emotional part, corresponding to the dimensions in GamTest (23). The application of this psycho-social model specification enables an exploration of over consumption as an explanatory variable for problematic use of games rather than just charting peripheral components, in accordance with the Charlton and Danford suggestion that the peripheral criteria might precede the core criteria (5, 23).

International studies have found the prevalence of disordered gaming to range between 1.3 and 6.8 percent (24). Stevens et al. report that the prevalence of disordered gaming worldwide in a meta-analysis is 3.05 percent (25). The differences in prevalence are likely due to differences in assessment methods, sample characteristics, and cultures in different countries (24, 26). Child and adolescent psychiatrists as well as school health care workers have reported disordered gaming among their patients and students. These clinicians describe compulsion, psychiatric and physical symptoms and impaired school performance as components of the disorder (27, 28). Most research on disordered gaming reports that males are more likely than females to experience disordered gaming (25, 27, 29–31) and the prevalence rates are commonly higher in adolescent samples (24, 25). Several previous studies report on the association between ADHD and disordered gaming (29, 32, 33) and DSM-5 lists ADHD as a comorbidity of IGD (1). Stavropoulos et al. presented a theory on gender dependent ADHD characteristics as a possible explanation to the gender discrepancy regarding disordered gaming (29). However, sex differences in gaming and potential gender specific health correlates are poorly understood.

In summary, the GASA is an established measure of gaming behavior, but the psychometric properties of the scale have previously mainly been investigated in adult or adolescent populations (16, 24, 25). Male gender and ADHD are frequently reported as risk factors for disordered gaming (24, 25, 29, 32, 33) but no previous research has to our knowledge evaluated how these factors relate to the components in GASA. This study contributes to the knowledge of gaming, using a clinical sample of children and adolescents to explore the psychosocial dimensions of the GASA.

The present study evaluates the indicators of gaming behavior in GASA from a multifactorial perspective and explores the gender differences in a clinical setting, focusing on ADHD. Both the two-factor core approach and an alternative three-factor version are analyzed psychometrically. The study aims are specified as follows:

1. Explore the dimensionality of the items in GASA and the potential impact of gender and/or ADHD.
2. Analyze the fitting of the two-factor core approach on the CAP sample.
3. Analyze the fitting of an adapted three-factor version of the core approach on the CAP sample, by dividing the core items into social and emotional categories.

## Methods

### Participants

The study was performed in Skane, a county in the south of Sweden with 1.36 million inhabitants, of which 280,000 are individuals under 18 years of age. In 2018 CAP Skane had 55,000 unique visits. There are seven out-patient child and adolescent psychiatry units in Skane and one in-patient unit. The out-patient units cater for all types of child and adolescent diagnoses but have no assignment to either diagnose or treat addiction problems. In the present study, patients coming to the Child and Adolescent Psychiatry clinic (in- and out-patient departments, respectively) in Skane during the study period of 4 months (Feb–May) during 2020 were asked to participate. Clinicians (psychologists, psychiatrists) were systematically provided with questionnaires and were asked to distribute these to their patients. The study was approved by the Ethics committee (Dnr: 2019-02967). Written informed consent was obtained from all participants and their parents/guardians.

The survey was answered by 144 children and adolescents between 8 and 18 years of age. Six individuals participated without sharing social security number which made the collecting of other information (gender, age, diagnosis) impossible. One individual abstained from answering the GASA-items. Concordantly, seven individuals were excluded from the data file leaving 137 individuals, characteristics specified in Table 1. The gender distribution was even, most of the participants were recruited through outpatient care and a majority were older than 13 years. The mean age was 14.5 years. The participant's main as well as secondary diagnosis, when applicable, was registered. The diagnoses were referred to as the Manual of Mental Disorders, 5th edition, describes them (1). ADHD was the most prevalent diagnosis. Other diagnoses that occurred were depression, autism spectrum disorder, anxiety, eating disorder, anxiety/depression, bipolar disease, obsessive compulsive disorder, social phobia, and psychosis. All patients were assessed in clinical settings by trained psychologists and child and adolescent psychiatrists.

**Table 1:** Descriptive statistics for CAP sample, *n* = 137.

**Description**	***N***	**%**
**Gender**		
Male	69	50.4
Female	68	49.6
**Type of care**		
Outpatient care	121	88.3
Inpatient care	16	11.7
**Age, years**		
8–12	28	20.4
13–18	109	79.6
**ADHD lifetime**		
Yes	57	41.6
No	80	58.4

Concerning sample size for the GASA analyses, the Price guidelines are for a minimum sample size equal to 105 (7 items × 15 patients) (34). The CAP sample includes *n* = 137 observations and accordingly fulfills the requirements according to guidelines (34).

### Measures

One of the most used questionnaires for disordered gaming in adolescents is GASA (Game Addiction Scale for Adolescents), constructed by Lemmens et al. (13–17). The seven-item GASA applies to gaming behavior in the last 6 months, see Table 2. Each item concerns one criterion, answered on a five-point scale: 1 = never, 2 = rarely, 3 = sometimes, 4 = often, 5 = very often and should be considered endorsed when rated 3 or higher (15).

**Table 2:** GASA, peripheral and core items corresponding to OC and NC, respectively.

**How often in the last 6 months:**	**Peripheral items**	**Core items**	**Addiction criterion**	**Early signs of problems^a^**
1. Have you thought all day long about playing a game?	x		Salience/preoccupation	OC
2. Have you played longer tan intended?	x		Tolerance	OC
3. Have you played games to forget about real life?	x		Mood modification	OC
4. Have other unsuccessfully tried to reduce your time spent on games?		x	Relapse	NC social
5. Have you felt upset when you were unable to play?		x	Withdrawal	NC emotional
6. Have you had arguments with others (e.g., family, friends) over your time spent on games?		x	Conflict	NC social
7. Have you neglected important activities (e.g., school, work, sports) to play games?		x	Problem/Neglect duties	NC emotional

a *According to GamTest (28, 29)*.

*GASA, game addiction scale for adolescents; OC, over consumption; NC, negative consequences*.

With support from previous research, empirical data and theoretical reasoning (5, 13–15, 19, 23, 35, 36) the GASA items were associated with the different factors in a psycho-social conceptual model to enable testing of a two-factor approach (core approach) and a three-factor approach, in which the peripheral items/negative consequences were differentiated into negative consequences social and negative consequences emotional, see Table 2. The psychosocial conceptual model is guiding the specification of measurement and structural models analyzed. For details see Supplementary Diagram 1 and paragraph 3 (conceptual model) and 4 (GASA instrument) in the electronic supplement. This model specification aims to consider over consumption as an explanatory variable for problematic use of games rather than a peripheral component.

The following variables were obtained from subjects in the study: GASA, gender, age, housing situation (with whom you live), type of care given at CAP (in-/out-patient care) and diagnosis at CAP.

### Statistical Analysis

Psychometric analyses including confirmatory factor analyses (CFA) were used to identify constructs captured by the GASA items through well-fitting measurement models. These analyses were performed within the latent variable framework in Mplus software Version 8.6 (30). Robust maximum-likelihood estimation MLR was applied to adjust for skewed item distributions in the goodness-of-fit testing. Item analysis and trimming of skewed item distributions was performed to improve the fulfillment of the requirements of the chi square testing in the CFA and SEM analyses Gender differences in GASA measurement models were assessed using multiple-group confirmatory factor analysis (MGCFA). In order to explore if the latent variables were equivalent across groups, test for invariance in measurements were executed for group comparisons of CFA models. Factor analysis of multiple groups considers three degrees of measurement invariance: configural, metric (also referred to as weak factorial invariance) and scalar (strong factorial invariance). In the present study, a two-group two factor metric model corresponding to the core approach shows acceptable fit. This measurement model with equality constraints for corresponding measurement models (metric invariance) across gender was used as the outcome variable in a multiple-group structural equation model (SEM) to examine gender differences exploring direct and indirect effects of a diagnosis of ADHD on over consumption and negative consequences social and emotional (34). Details are available in the electronic supplement.

Goodness of Fit Indexes were calculated for the One- Two- and Three factor Solutions to the GASA Scale, for the whole sample (*n* = 137) and divided according to gender (male *n* = 69, female *n* = 68), with metric invariance, with and without equality constraints. Ever being diagnosed with ADHD was added as a covariate independent variable, hereafter mentioned as ADHD lifetime. The goodness-of-fit of the CFA/SEM models was assessed using the root mean square error of approximation (RMSEA) and the comparative fit index (CFI). Values above 0.95 (CFI) and below 0.08 (RMSEA) were considered acceptable (37, 38).

A three-factor model in which negative consequences was differentiated into social and emotional harm was explored regarding the impact of over consumption.

This measurement model (3.1 gm) was used as a vehicle to test gender differences exploring direct and indirect effects of the risk factor diagnose ADHD lifetime on over consumption and negative consequences social and negative consequences emotional (Model 3.2 gdia).This model assumed equality constraints for corresponding measurement models, see Table 3.

**Table 3:** Goodness of fit Indexes for the one-, two- and three-factor solutions of GASA.

**Model**	**Model description**	**CFI**	**RMSEA**
1.1	GASA CFA 1 core items NC all	0.994	0.051
1.1 g	GASA MGCFA 1 core items NC by gender configural	0.954	0.077
1.2	GASA CFA 1 OC and NC all	0.960	0.077
1.2 g	GASA MGCFA 1 OC and NC by gender configural	0.886	0.095
2.1	GASA CFA 2 all	0.973	0.065
2.1 g	GASA MGCFA 2 by gender configural	0.933	0.077
2.1 gc	GASA CFA 2 by gender configural with correlation errors between item 5 and 7	0.971	0.059
2.1 gm	GASA MGCFA 2 by gender metric, model 2.1 gc with eq constraints	0.935	0.079
3.1	GASA CFA 3 all	0.974	0.069
3.1 gm	GASA MGCFA 3 by gender metric eq constraints	0.959	0.069
3.2 g.dia	GASA MCCFA 3 by gender model 3.1 gm with covariate diagnose ADHD lifetime	0.954	0.067

*Whole sample all n = 137 and multiple-group by gender, n (female) = 68, n (male) = 69. CFI, comparative fit index; RMSEA, root mean square error of approximation; GASA, game addiction scale for adolescents; CFA, confirmatory factor analyses; MGCFA, multiple-group confirmatory factor analysis; OC, over consumption; NC, negative consequences; ADHD, attention deficit hyperactivity disorder*.

Factor scores and means optimized for measurement non-invariance across gender were computed with the alignment procedure in Mplus based on a one-factor model fitted to over consumption items and negative consequences items separately (39). Factor scores and means in an alignment optimization metric were saved for further post processing in SPSS, for details see Supplementary Table 4 in the electronic supplement. ANOVA testing of effects by gender and ADHD lifetime diagnosis as well as gender and age group were reported with the test variable F. All statistical analyses are based on the reduced sample *n* = 137, with no missing data. Details are available in the electronic supplement.

## Results

### Overview of Goodness of Fit Results for GASA CFA and SEM Models

Model fitting results are reported in Table 3. The Goodness of Fit Indexes for models including all 7 items, the one-factor model (model 1.2) and the two-factor model/core approach (Model 2.1), showed a good model fit. The Goodness of Fit index for the two-factor model did not meet the cutoff values when the sample was divided by gender. When correlation errors between item 5 and 7 and equality constraints were added the adjusted two-factor model showed an acceptable fit. The three-factor model (model 3.1) showed a good fit for the whole sample and when divided by gender and when being diagnosed with ADHD lifetime was added as a covariate diagnose.

### The Psychometric Model for the Core Approach

**Figure 1:**
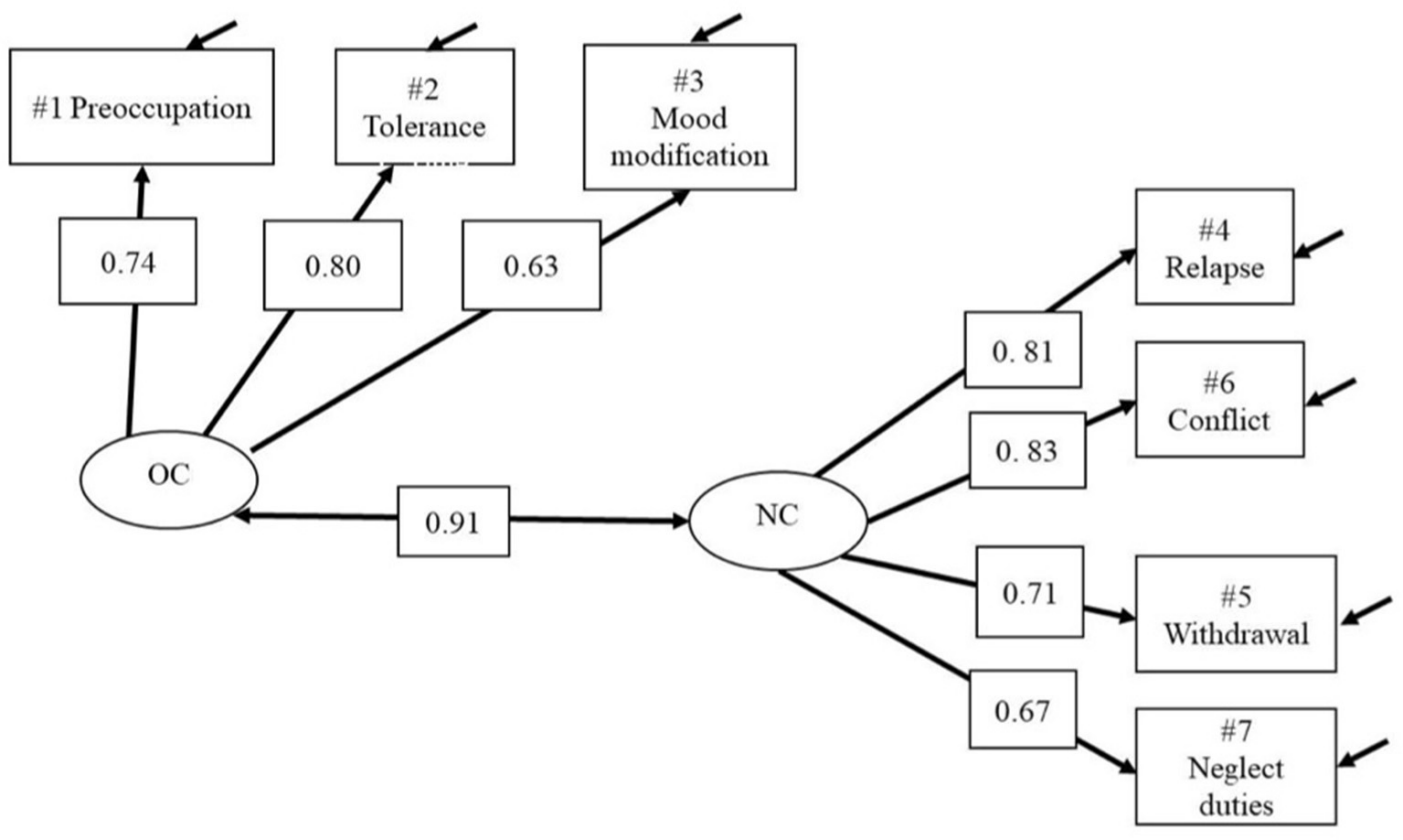
Model 2.1—GASA CFA 2 all. Two-factor Core approach model, OC/Peripheral and NC/Core. OC, over consumption; NC, negative consequences.

Path Diagram for the two-factor CFA model, peripheral-core approach is reported in Figure 1. The peripheral items correspond to over consumption (OC) and the core items reflect negative consequences (NC). In the measurement Model 2.1 (Figure 1) the estimate of the correlation between f (OC) and f (NC) was high, 0.91. The model showed an acceptable fit (CFI = 0.973; RMSEA = 0.065) which confirmed that the core approach shows a valid factor structure for the total sample *n* = 137 (see Figure 1; Table 3).

**Figure 2:**
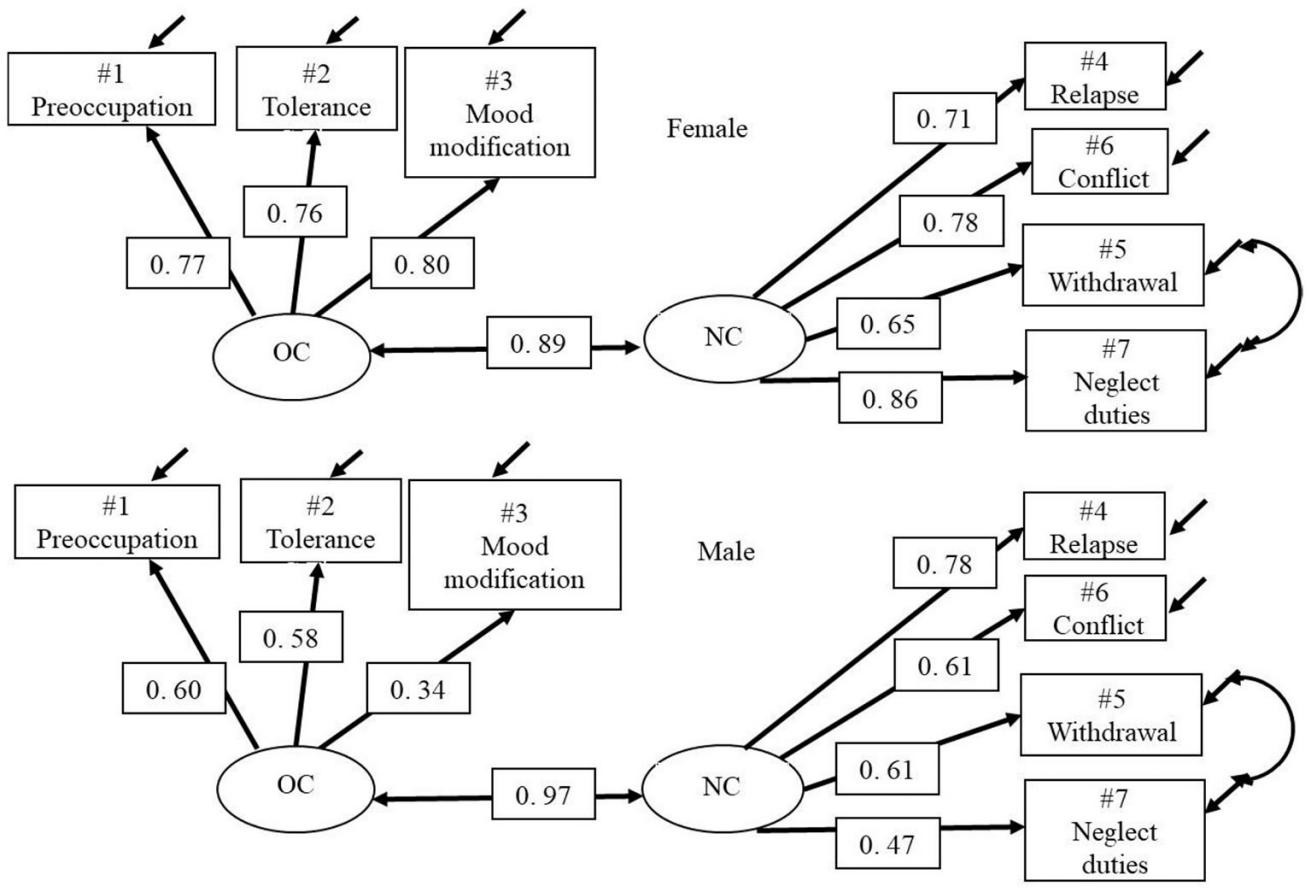
Model 2.1 gc—GASA MGCFA 2 by gender configural. Two-group two-factor Core approach model, OC/Peripheral and NC/Core. With correlated errors between NC item #5 Withdrawal and #7 Neglect duties.

The two-factor model (core approach) divided by gender showed a CFI value just below 0.95. When inserting the correlation between error terms for item 5 (withdrawal) and 7 (neglect duties) in Model 2.1 g (the negative consequences emotional factor) the goodness of fit was improved, see Figure 2 and Table 3. The correlation between OC and NC latent variables was 0.89 for girls and 0.97 for boys.

### The Three-Factor Model

**Figure 3:**
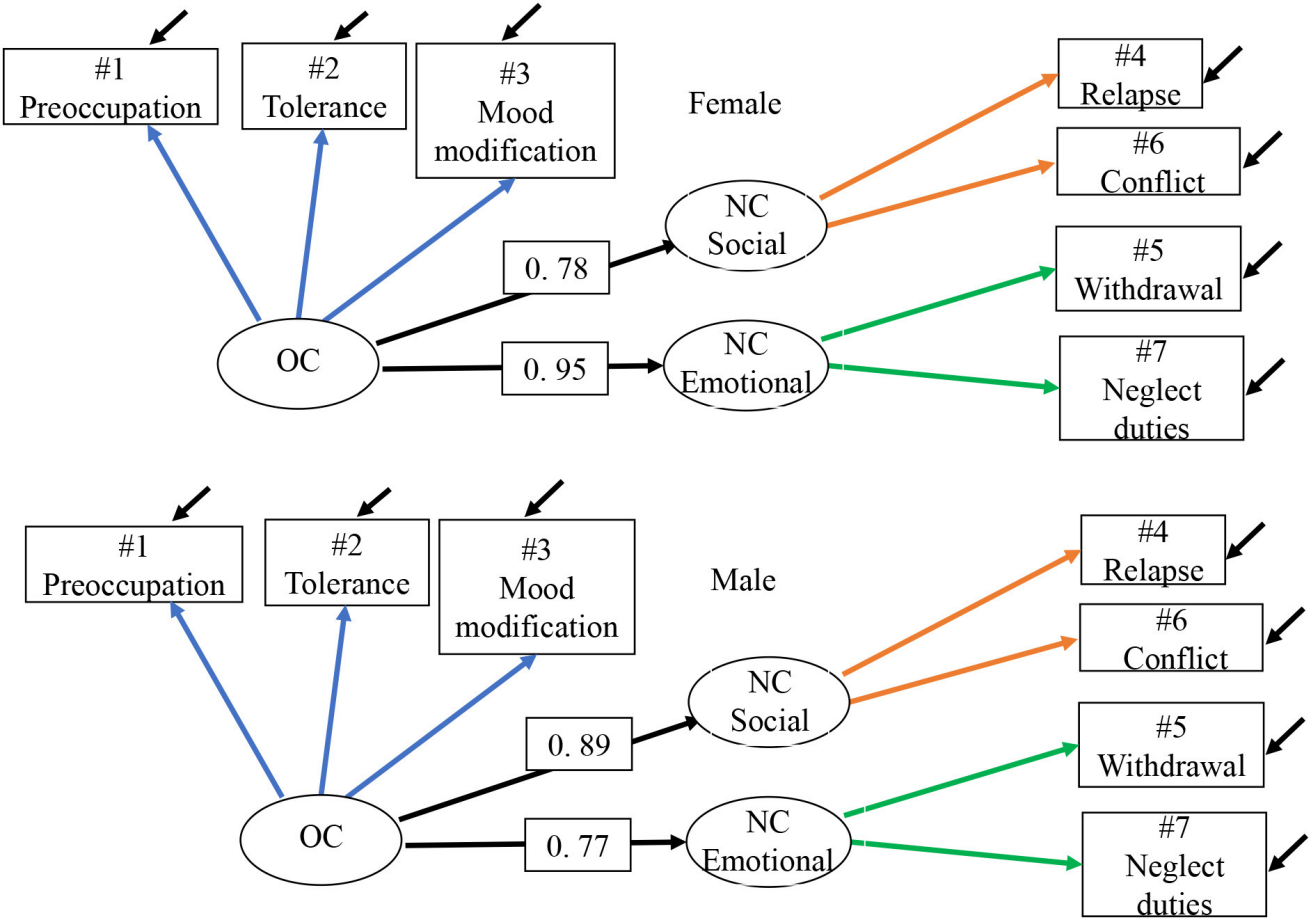
Model 3.1 gm—GASA MGCFA 3 by gender, metric. Two-group three-factor model by gender with core items divided into NC social and emotional with equality constraints across gender groups for corresponding measurement models. Residual correlations NC social with NC emotional (not represenated in the path diagram) for males is 0.40 and for females 0.87. OC, over consumption; NC, negative consequences.

The three-factor model showed an acceptable fit (CFI = 0.974; RMSEA = 0.069) which confirms that this alternative version of the core approach constitutes a valid factor structure for the total sample *n* = 137. The factor structure remained valid when analyzed with a two-group model with equality constraints across gender groups for corresponding measurement models (see Table 3; Figure 3).

Residual correlations of NC social with NC emotional (not represented in the path diagram) for males was 0.40 and for females 0.87. When the path coefficient for OC → NC was differentiated into a social and emotional path coefficient, the strongest relationship for boys appeared as OC → NC social equal to 0.89 and for girls OC → NC emotional equal to 0.95.

### Gender Differences in the Three-Factor Model With Covariate ADHD

**Figure 4:**
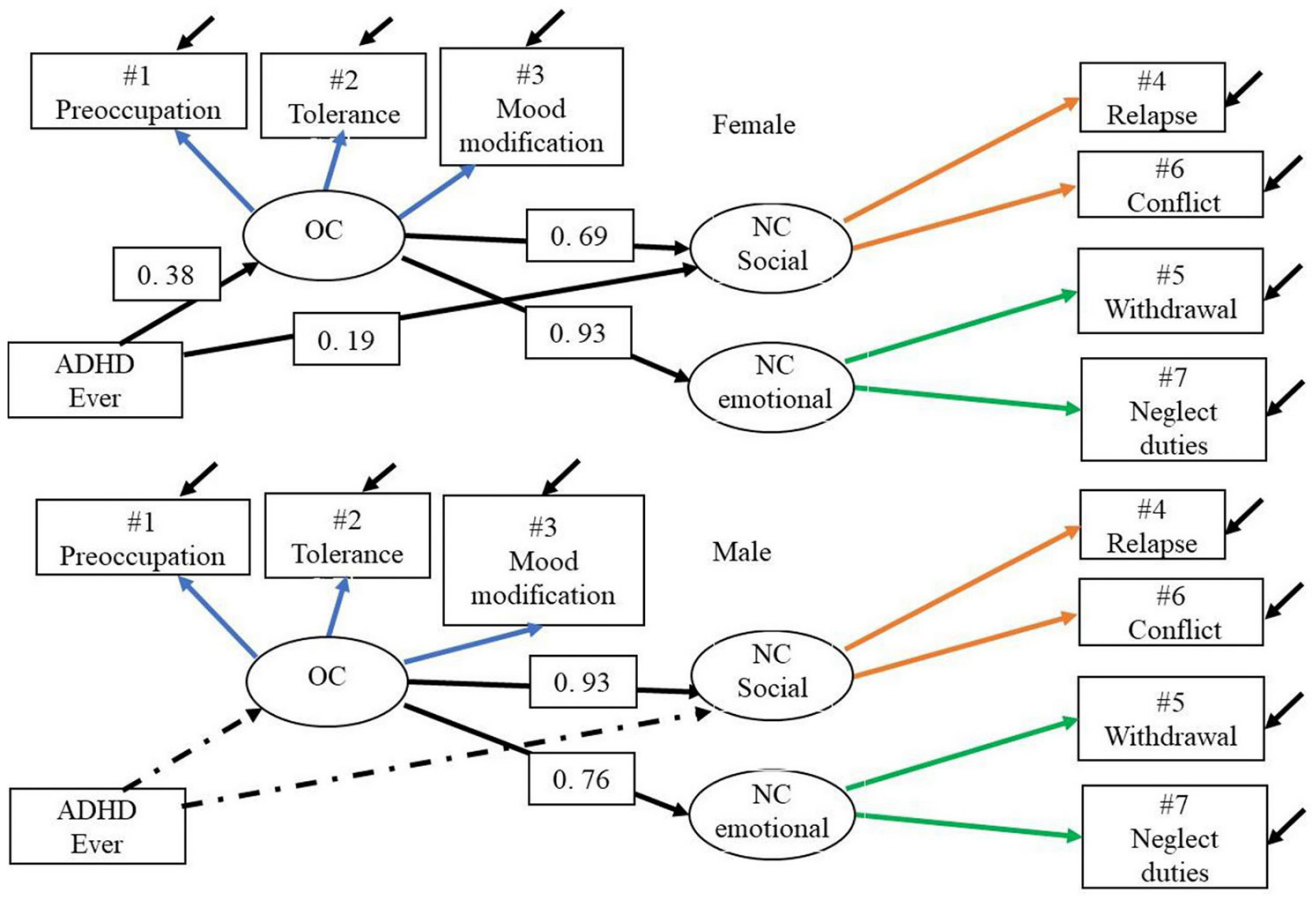
Model 3.2 g.dia—GASA MGCFA 3 by gender, metric with covariate. The two-group three-factor model with equality constraints across gender for corresponding measurement models and with covariate ADHD ever. Dotted line is non-significant path. OC, over consumption; NC, negative consequences.

When the risk factor being diagnosed with ADHD was added as a covariate the estimated path coefficient showed that ADHD constituted a significant correlate for both over consumption of gaming and negative consequences specified as social for females but not for males, see Figure 4.

### The Impact of Age and ADHD on Differences in Gaming Behavior for Boys and for Girls

**Figure 5:**
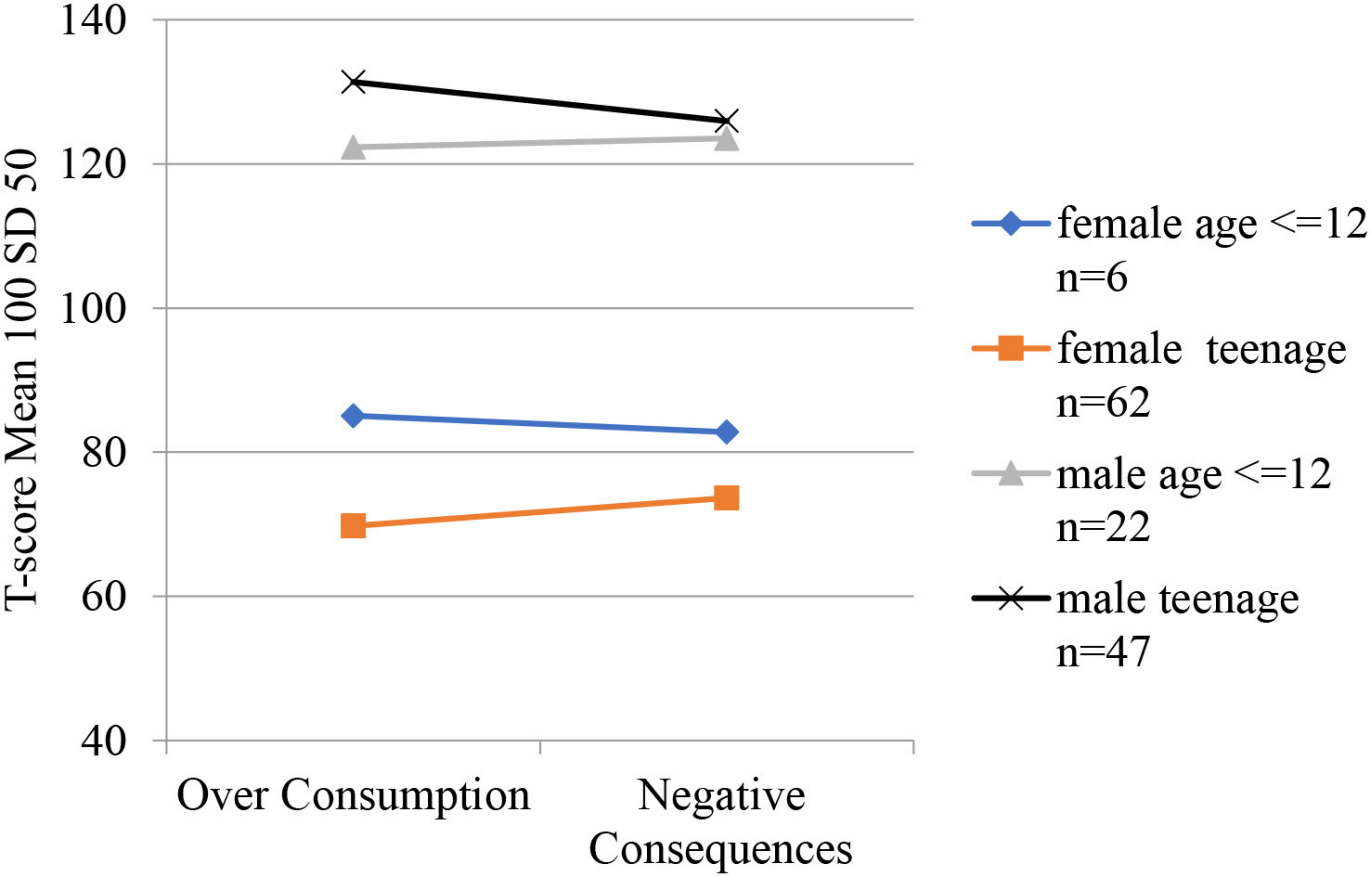
Over consumption and negative consequences mean profiles for gender by age groups. Scale is aligned factor T-scores. Mean 100 and SD 50 for the CAP sample. For data see electroninc Supplementary Table 6.

**Figure 6:**
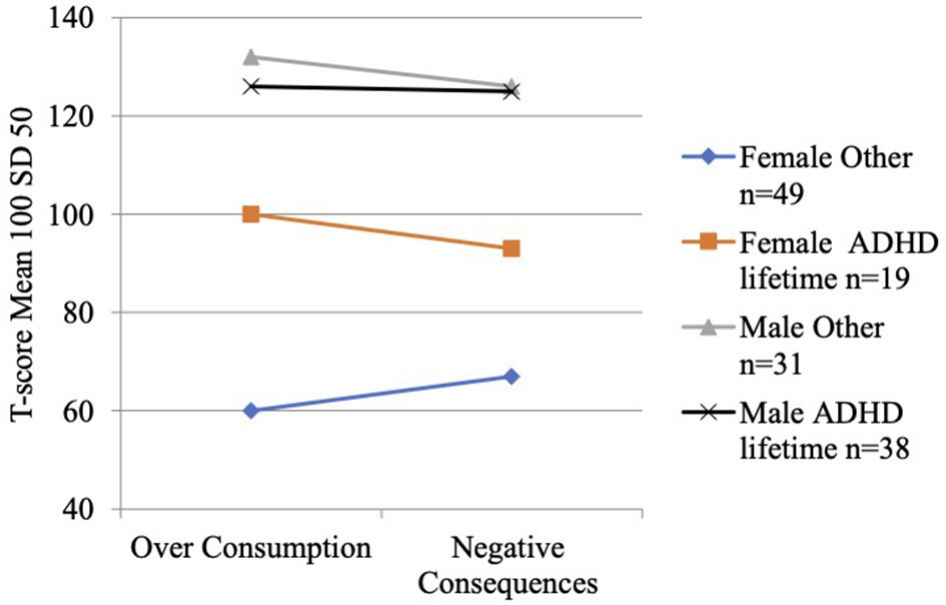
Over consumption and negative consequences mean profiles for gender by ADHD lifetime diagnosis groups. Scale is aligned factor T-scores. Mean 100 and SD 50 for the CAP sample. For data see electroninc Supplementary Table 6.

The new aligned T-scores measure severity of over consumption and negative consequences at a common scale. Minor and non-significant differences appeared between the child and teenage groups concerning their gaming severity, both regarding over consumption and negative consequences, among both male and female participants. The effect of age is illustrated in Figure 5 and further described in ANOVA tests reported in Supplementary Table 8 and through descriptive statistics in Supplementary Table 9 in the electronic supplement. The female participants show a significant difference between ADHD lifetime and other diagnoses both for over consumption (mean 100 vs. 60, *p* = 0.01) and for negative consequences (mean 93 vs. 67, *p* = 0.03) while male's mean profiles are very close and non-significant but at a higher level compared with the females. The interaction effect is illustrated in Figure 6 and further described in ANOVA tests reported in Supplementary Table 5 and through descriptive statistics in Supplementary Table 6 in the electronic supplement.

## Discussion

The present study contributes to our understanding of the dimensionality of GASA but also presents results that indicate a gender dependent distinction regarding the negative consequences of over consumption of gaming. The two-factor model of the core approach showed a satisfactory fit to the data. The three-factor version of the core approach also showed a good fit, when differentiating the negative consequences core items into social and emotional consequences. Interestingly, our findings suggest that over consumption of video games is more heavily associated with negative consequences for male gamers but also that their negative consequences of over consumption tend to be social rather than emotional, as was the case for female gamers. ADHD was significantly associated with over consumption of video games and the negative consequences thereof for girls. The male participants over consumed games to a higher degree than the females and showed more severe consequences, regardless of a potential ADHD diagnosis.

The fact that the three-factor model showed a good fit to the data confirms that the division of negative consequences into negative consequences social and negative consequences emotional could be a valid alternative factor structure. However, these constructs only contain two items each, making the social and emotional dimensions insufficiently grounded for reliable factor scores. Three items with high loadings are required to establish a solid factor (34). This suggests that further development of GASA is needed in order to capture both social and emotional components.

Most research agree that male gender is a risk factor for disordered gaming (25, 27, 30). Boys in general tend to spend more time on gaming and they are overrepresented among the minority that exhibits gaming problems (27, 30). Time spent on gaming has been reported as a risk factor for disordered gaming (30, 40) but whether the time spent constitute a greater risk for boys than for girls remains unclear. Our findings suggest that the association between over consumption of games and negative consequences thereof is stronger for boys. Further, our results suggest that the negative consequences of over consumption take a social direction for boys and an emotional direction for girls, a distinction that warrant additional investigation. Bonnaire et al. investigated gender differences in disordered gaming and showed that male gamers were disproportionately more likely to be single than female gamers whereas the female gamers showed a higher anxiety score (31). Possibly, the results presented by Bonnaire et al. (31) supports the tendency shown in this study using the three-factor structure of GASA, illuminating gender distinctive emotional and social consequences of gaming. GASA could be further developed with complementary items on social as well as emotional aspects of gaming in order to determine and further explore a potential psychosocial gender discrepancy of disordered gaming. In the 15-item gambling Gam Test the emotional factor was measured with 5 items, including aspects such as; feeling bad when thinking about gambling, gambling resulting in feelings of irritation and “I do not want to tell other people about how much time and money I spend on my gambling” (23). Similar items, adapted to gaming and to young individuals, could theoretically be added to the GASA to strengthen the factors of both the emotional and social dimensions.

ADHD is one of the most prevalent neurodevelopmental disorder in childhood with an estimated prevalence of 5 per cent, globally (41, 42). It is a heterogeneous condition with persistent symptoms of hyperactivity, inattention and impulsiveness that impair functioning in multiple settings (1). Researchers have found that ADHD is a particular risk factor for disordered gaming (32, 33, 43–46). In the current CAP sample, ADHD was significantly associated with over consumption of video games and the negative consequences thereof for girls, an association that was not seen among the male participants. Possibly, our results could be interpreted as being diagnosed with ADHD increases the risk of over consumption of computer games and the negative consequences thereof more for girls than for boys. To our knowledge, this gender discrepancy has not previously been explored. However, consistent with our findings, Yen et al. showed that the association between ADHD and Internet addiction was greater among female than male college students (32). Somewhat contractionary to our findings, Stavropoulos et al. hypothesized that the fact that female ADHD predominantly demonstrates inattention while males rather experience hyperactivity-impulsivity symptoms could contribute to a gender discrepancy regarding disordered gaming (29). They further hypothesized that hyperactivity-impulsivity mediates a greater risk for disordered gaming, which they managed to demonstrate, in consistency with other research (29, 47). However, Stavropoulos et al., did neither investigate whether ADHD is associated with a greater increase in risk for disordered gaming for either boys or girls nor did they define whether female gender affected the impact of hyperactivity-impulsivity/inattention. Martins et al., who investigated gender differences in mental health characteristics among adolescent gamblers, showed that parents to female gamblers were disproportionally likely to rate high levels of childhood hyperactivity when compared to parents to male gamblers (48). Since both gaming and gambling are more common and socially accepted behaviors among men, it is possible that women are more prone to exhibit predisposing conditions. Regardless, our findings warrant additional research to establish and explain a potential gender discrepancy regarding the association between ADHD and disordered gaming.

### Strengths

The study provides an interdisciplinary perspective on diagnostic testing and applies a psychometric methodology capable of uncovering different aspects of gaming behavior in a clinical setting (49). Specifically, the statistical analyses take measurement errors in criteria as well as sample size into account. Alternative measurement models are tested for goodness-of-fit, including test for invariance across gender groups (34). In summary, the methodology is grounding the results in qualified empirical evidence.

### Limitations

The present study does have some limitations. One limitation is the cross-sectional design which does not allow for conclusions regarding cause and effect. In order to explore causation a longitudinal investigation is required. Further, the measures used for this study are partly based on self-reporting, which implies a risk for recall bias. One other limitation is a possible selection bias. Clinicians were provided with questionnaires and were supposed to distribute them to their patients, but the study design does not provide any insight into the numbers of patients declining or more importantly why. However, the gender distribution was even, ADHD was the most prevalent disorder, as expected (41, 42) and we have no obvious reason to believe that the sample excelled heavily from an ordinary CAP population. The different specifications of alternative models relating over consumption with negative consequences show that the relationship is remarkably high, with correlations as high as 0.97, possible reflecting a weakness in the self-test of a strong general method factor present as part of both over consumption and negative consequences. Among issues in the design of GASA and in data collection causing bias in the correlation between over consumption and negative consequences through such a factor, is low motivation for youth to engage in answering questionnaires (50). Furthermore, GASA was originally developed based on the DSM-5 criteria for pathological gambling (15). Disordered gaming behavior among youth may involve other issues than those involved in gambling among adults.

## Conclusion

The psychometric approach differentiates information gathered using established diagnostic instruments like GASA into measures of behavior lying underneath the different markers/diagnostic criteria. Available diagnostic instruments could be strengthened by complementary items designed for children and youth in order to illuminate the complexity of gaming behavior. Our results suggest that the association between over consumption of games and negative consequences thereof is stronger for boys than for girls. Negative consequences of over consumption take a social direction for boys and an emotional direction for girls. ADHD was significantly associated with over consumption of video games and the negative consequences thereof for girls, an association that was not seen among the male participants. Together, our findings should encourage further developments of the GASA instrument and additional research on potential gender related discrepancies of disordered gaming.

## Data Availability Statement

Data can be made available in case of a formal request from the authors to the ethics committee. Requests to access the datasets should be directed to: anders_c.hakansson@med.lu.se.

## Ethics Statement

The studies involving human participants were reviewed and approved by Swedish Ethics Committee (Dnr: 2019-02967). Written informed consent to participate in this study was provided by the participants' legal guardian/next of kin.

## Author Contributions

FA: investigation, visualization, software, methodology, conceptualization, and writing. IM: data curation, visualization, software, methodology, conceptualization, formal analysis, and writing. AH: validation, methodology, conceptualization, writing, project administration, and supervision. EC-K: investigation, visualization, software, methodology, conceptualization, writing, resources, and supervision. All authors agree to be accountable for the content of the work.

## Funding

This study was funded by the Svenska Spel Research Council, Fanny Ekdahls Foundation, FoURegional funds of Region Skane, SUS funds and stipends, Craaford foundation, and Sigurd and Elsa Goljes memorial fund. None of these bodies had any role in, or influence on, the present study. The authors alone are responsible for the content and writing of the paper.

## Conflict of Interest

AH holds a position at Lund University which is sponsored by the state-owned Swedish gambling operator Svenska Spel and also has research funding from the research council of the Swedish state monopoly for alcohol, Systembolaget AB. The remaining authors declare that the research was conducted in the absence of any commercial or financial relationships that could be construed as a potential conflict of interest.

## Publisher's Note

All claims expressed in this article are solely those of the authors and do not necessarily represent those of their affiliated organizations, or those of the publisher, the editors and the reviewers. Any product that may be evaluated in this article, or claim that may be made by its manufacturer, is not guaranteed or endorsed by the publisher.
