# Supplementary material for: Game Addiction Scale for Adolescents—Psychometric Analyses of Gaming Behavior, Gender Differences and ADHD
Source: Front Psychiatry. 2022 Mar 9;13:791254. doi: 10.3389/fpsyt.2022.791254 (PMC8959768; doi:10.3389/fpsyt.2022.791254)
Supplement: Supplementary file 1 [file Presentation_1.pdf]

Supplement. Game addiction scale for adolescents (GASA) - psychometric analyses of gaming behavior. gender differences and ADHD.

DOI: 10.3389/fpsyt.2022.791254

Frida André[1] Ingrid Munck[2]

[1]Lund University, Faculty of Medicine, Department of Clinical Sciences Lund, Sweden.

[2] University of Gothenburg, Department of Education and Special Education, Göteborg, Sweden

This report is a supplement to the frontier Psychiatry article " Game addiction scale for adolescents (GASA) - psychometric analyses of gaming behavior and gender differences" by Frida André, Ingrid Munck, Anders Håkansson, and Emma Cleaesdotter-Knutsson, 2022 Vol 13 article 791254, available on-line for complementary information about primary the conceptual model and the statistical analyses. The data preparation is performed in SPSS version 27 and the psychometric and gender difference analyses in software Mplus version 8.6 (Muthén and Muthén 2020).

Content

1. Introduction
2. Objectives
3. Conceptual model
4. The GASA instrument
5. Data and file building
6. Statistical analyses
7. New GASA measurement models
8. Two applications
9. Epilog
10. References

Appendix GASA Psychometric analyses - Mplus Input and Output.

1. Introduction

Paper I of the CAP Skåne study applied the core approach to the analyses of the GASA self-test and pointed at gender as the overall most influential risk factor on gaming problems (André et al 2021; André and Munck 2021). This article is a further analysis of the CAP sample with psychometric analyses. It introduces a multidimensional perspective, measuring the different aspects of gaming behavior and harm captured by the Game Addiction Scale for Adolescents (GASA) 7 item instrument. A theoretical framework of the different factors to consider in researching the development of gaming behavior and the problematic use of games and its consequences is outlined.

Psychometric analysis will identify various constructs based on psychological and social risk factors for gaming behavior. Gender differences in such composites will be explored and further analyzed with structural equation models (SEM). The sample contains n=69 male and n=68 female cases.

The software Mplus version 8.6 (Muthén and Muthén 1998-2020) is used for CFA and SEM analyses. This program for statistical analyses with latent variables has the capacity of advanced measurement analyses to differentiate the information contained in established diagnose instruments like GASA and uncover what kind of behavior is behind the different criteria from a multifactorial perspective (Rupp 2010).

2. Objectives

The overall aim is to develop multi factorial models for the GASA self-test and identify well-defined constructs grounded in a psycho-social conceptual model for gaming behavior. Hypnotized constructs for gaming behavior and harm are Overconsumption and Negative consequences corresponding to Peripheral and Core factors, respectively.

Another goal is to demonstrate how these new composite measures of gaming behavior are related to risk and protection factors and test differences between gender groups.

With psychometric methods we will uncover well-defined constructs of gaming problems and test multi factorial models applied to the clinical CAP sample (André et al 2021; Price, L.R. 2017). Two-group analyses of gender differences will show if measures of gaming problems are comparable across female and male. Factor scores and means optimized for measurement non-invariance across gender are computed with the alignment procedure in Mplus (Asparouhov and Muthén 2014). These aligned factor scores are refined new gaming problem variables for further descriptive statistics and ANOVA testing of effects by age, gender, and diagnoses.

3. Conceptual model

In searching for some theoretical bases for a conceptual framework for gaming behavior and harm let's turn to a recent study by Jonsson (2019) that put forward some theoretical assumptions concerning gambling problems and discuss which are relevant also for gaming among youth.

Quote page 57 in Jonsson 2019:

This thesis is based on the following theoretical assumptions.

a. Gambling problems can be seen as a continuum, with gambling without problems at one end. One can have mild to severe gambling problems, and an individual can move back and forth along the continuum. Also, it is meaningful to have different preventive measures at different levels of gambling and gambling problems.

b. The aetiology/origin of problem gambling is multifactorial, with various risk factors from the biological, psychological and social domains. Most problem gamblers have more than one or two risk factors, and they are probably additive in increasing risks for gambling problems. Some patterns of risk factors among problem gamblers are more common (as for example suggested by the Pathway Model developed by Blaszczynski and Nower (2002) proposed three subgroups of gamblers with impaired control: (1) behaviorally conditioned, (2) emotionally vulnerable and (3) antisocial, impulsive problem gamblers. A comprehensive

pathway model leading to problem gambling is presented with a focus on psychopathology and gambling motives.

c. Gambling can be psychologically rewarding in various ways. If the gambling is strongly rewarding for the individual, it constitutes a risk factor for overconsumption.

d. Overconsumption of gambling is a precursor of gambling problems and can be seen as an early stage of loss of control. …………………… End of quote.

Based on assumption that these theoretical considerations also are relevant for youth in Diagram S1 a conceptual model for gaming behavior and harm is outlined to describe the different factors to consider in researching the development of gambling or gaming behavior and the problematic use of games and its consequences.

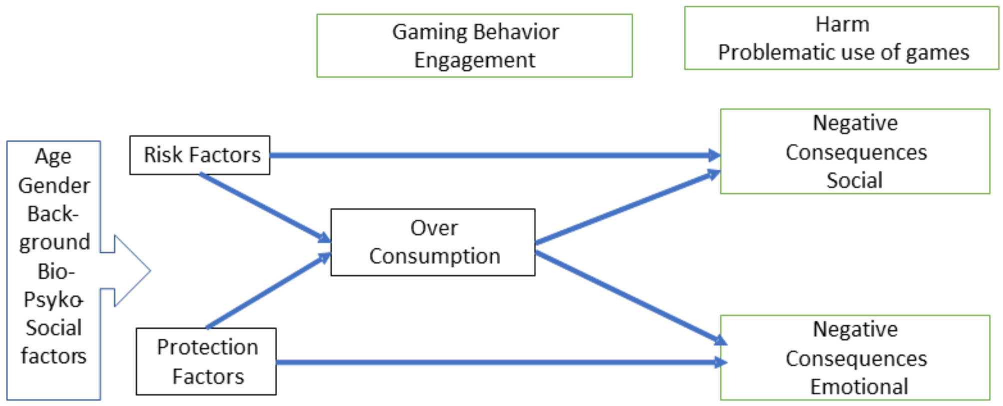

Diagram S1. A Psycho-Social Conceptual Model for Gaming Behavior and Harm.

4. GASA instrument

For the assessment of gaming behavior, the seven-item version of the Gaming Addiction Scale for Adolescents (GASA) instrument is used. The scale was constructed by Lemmens et al. to reflect components of addiction as well as the consequences thereof, namely: salience, tolerance, mood modification, relapse, withdrawal, conflict, and problems. Each question covers one criterion, answered on a five-point continuum scale: 1 (never), 2 (rarely), 3 (sometimes), 4 (often), 5 (very often) and should according to the developer be accounted as endorsed when rated 3 or higher (Lemmens et al., 2009, 2015).

Brunborg et al 2015 two factor model – the core approach based on the GASA self-test.

Aiming to distinguish level of severity within the group of gamers, a two-factor structure, which separate peripheral criteria from core criteria, has been shown to fit in a survey of Norwegian aged 16-74years (Brunborg et al 2013, 2015).

In the conceptual framework presented in Diagram S1 the Peripheral factor, items # 1, #2 and #3, correspond to the construct OC while Core factor, items where #4 Relapse and #6 Conflict both concern 'others' or a social component of NC and #5 Withdrawal and #7 Neglect duties are psychological indicators of emotional NC. In Table S1 the different addiction criteria for gaming covered by the GASA instrument are reported and which items correspond to the core

and peripheral construct respectively, with reference to the conceptual model for gaming behavior and harm in Diagram S1a.

Table S1a(Table 1 in article) GASA, peripheral and core items corresponding to OC and NC respectively

How often in the last six months:	Peripheral items	Core items	Addiction criterion	Early signs of problems[a]
1. Have you thought all day long about playing a game?	x		Salience/ Preoccupation	OC
2. Have you played longer than intended?	x		Tolerance	OC
3. Have you played games to forget about real life?	x		Mood modification	OC
4. Have other unsuccessfully tried to reduce your time spent on games?		x	Relapse	NC social
5. Have you felt upset when you were unable to play?		x	Withdrawal	NC emotional
6. Have you had arguments with others (e.g., family, friends) over your time spent on games?		x	Conflict	NC social
7. Have you neglected important activities (e.g., school, work, sports) to play games?		x	Problem/Neglect duties	NC emotional

Notes: [a]According to GamTest Jonsson et al 2017.

Abbreviations: GASA, game addiction scale for adolescents; OC, over consumption; NC, negative consequences.

5. Data and file building

Data

The survey was answered by 144 children and adolescents between 8 and 18 years of age. Six individuals participated without sharing social security number which made the collecting of other information (gender, age, housing situation, type of care, diagnosis) impossible. These 6 individuals were excluded from the data file together with one individual abstained from answering the GASA-items leaving 137 remaining individuals.

Sample size

Concerning sample size for the GASA analyses the guidelines according to Price 2017 are for a minimum sample size equal to 105 (7 items x 15 patients) considering principal component coefficients ranging from 0.69 – 0.83 (see Supplement Table 1c.). The CAP sample has

n=137 observations and fulfill therefore with marginal the requirements according to guidelines from a recent textbook.

Diagnoses

The participant's main diagnosis was registered and when considered relevant also a secondary diagnosis. The diagnoses were referred to as the Manual of Mental Disorders, 5th edition, describes them (APA 2013). All patients were assessed in clinical settings by trained psychologists and child and adolescent psychiatrists.

Frequency distributions and non-normality

The gender distribution was very even, n(female)=68 and n(male)=69.

The age distribution has mean 14.5 and median 15. A majority were older than 13 and the age 16 was overrepresented (22 per cent), whereas only one individual was 8 years old and one 18 years old.

Table S1b. CAP study Age distribution.

	Age years							
Statistics	Age <=11	12	13	14	15	16	Age >=17	Total
Frequency	15	13	14	18	22	30	25	137
Percent	10.9	9.5	10.2	13.1	16.1	21.9	18.2	100.0

The test item distributions are trimmed to be less skewed to adjust for non-normality.

SPSS recoding statements

```
RECODE GASA1 (MISSING=SYSMIS) (1=1) (2 thru 3=2) (4 thru 5=3) INTO GASA1r.
EXECUTE.
RECODE GASA3 (MISSING=SYSMIS) (1=1) (2 thru 3=2) (4 thru 5=3) INTO GASA3r.
EXECUTE.
RECODE GASA5 (MISSING=SYSMIS) (1=1) (2 thru 3=2) (4 thru 5=3) INTO GASA5r.
EXECUTE.
RECODE GASA7 (MISSING=SYSMIS) (1=1) (2 thru 3=2) (4 thru 5=3) INTO GASA7r.
EXECUTE.
RECODE GASA2 (MISSING=SYSMIS) (1=1)(2=2)(3=3) (4 thru 5=4) INTO GASA2r.
EXECUTE.
RECODE GASA4(MISSING=SYSMIS) (1=1) (2=2)(3=3) (4 thru 5=4) INTO GASA4r.
EXECUTE.
RECODE GASA6 (MISSING=SYSMIS) (1=1)(2=2)(3=3)  (4 thru 5=4) INTO GASA6r.
EXECUTE.
```

In Table S1c skewness is reported before and after recoding reducing skewness for all items in order to improve to fulfill the non-normal criteria for the chi square testing in the CFA and SEM analyses.

Table S1c.Descriptive Statistics for GASA items. Skewness reported before and after recoding.

GASA Item		GASA original scale			GASA scale after recoding	
#	Addiction criterion	Mean	SD	Skewness	Skewness	Reliability*
1	Salience/Preoccupation	2.34	1.369	0.614	0.310	0.76
2	Tolerance	2.35	1.246	0.533	0.236	0.79
3	Mood modification	2.28	1.494	0.749	0.486	0.69
4	Relapse	2.01	1.248	1.099	0.713	0.82
5	Withdrawal	1.77	1.226	1.429	1.204	0.77
6	Conflict	1.95	1.227	1.166	0.843	0.83
7	Problem/Neglect duties	1.85	1.192	1.239	0.876	0.73
	* SPSS procedure Dimension reduction Extraction Method: Principal Component Analysis One component					

Mplus dat-file contain the following variables:

Var list is ID Gender ADHD_lifetime Depression_lifetime ASD_lifetime Anxiety_lifetime Other_lifetime Teenage GASA1r GASA2r GASA3r GASA4r GASA5r GASA6r GASA7r.

6. Statistical analyses

SEM modelling, or covariance structure modelling, with latent variables has advanced more and more with less restrictions and robust estimators for non-normality features that are implemented in the applied software Mplus version 8.6. A rich literature of applications shows that the potential of SEM modelling is depending mainly on sample size, number of manifest variables and non-normality. The guiding textbooks used as references in this study are Muthén et al (2016) and Price (2017).

Concerning enough datapoints to estimate the parameters involved it is dependent on the covariance matrix between manifest variables, the 7 GASA items. Unless the model fixes sufficiently many parameters, the parameters are not identifiable with no unique estimates. The software Mplus is guiding about non-identified parameters which in this study's reported models has not been a problem.

Psychometric analyses including confirmatory factor analyses (CFA) are used to identify constructs captured by GASA through well-fitting measurement models. They are performed within the latent variable framework in Mplus software Version 8.6 applying robust maximum-likelihood estimation MLR to adjust for skewed item distributions in the goodness-of-fit testing. Gender differences in GASA measurement models are estimated with multiple-group confirmatory factor analysis (MGCFA).(Price, L.R. 2017; Asparouhov and Muthén 2009; Muthén and Muthén 1998-2020). The goodness-of-fit of the CFA/SEM models are evaluated using the root mean square error of approximation (RMSEA). Values smaller than 0.05 support a good model fit, while fit in the interval 0.06–0.08 is acceptable (MacCallum et al. 1996, Raykov et al. 2016).

In group comparisons of CFA models, it is important to explore if the latent variables are equivalent across groups, test for invariance in measurements. Factor analysis of multiple groups MGCFA considers three different degrees of measurement invariance: configural, metric (also referred to as weak factorial invariance) and scalar (strong factorial invariance). In the present study it will be shown that a two-group two factor metric model corresponding to the core approach has acceptable fit. This measurement model with equality constraints for corresponding measurement models (metric invariance) across gender is used as outcome variable in a multiple-group structural equation model (SEM) to test gender differences exploring direct and indirect effects of a diagnosis on OC and NC social and NC emotional (Price, 2017).

The strongest factorial invariance, scalar not achieved in this study, specifies that both the factor loadings and the measurement intercepts are invariant which makes it possible to compare factor means and factor intercepts across groups.

An alternative to compute factor scores adjusted for non-invariance for a configural model is the alignment procedure available in Mplus version 8 (Asparouhov and Muthén 2014; Muthén and Asparouhov 2018). Such aligned factor scores are refined new gaming problem variables for further descriptive statistics and ANOVA testing of effects by age, gender, and diagnoses. Factor scores from this model are saved for further statistical analyses in SPSS relating the new gaming severity variables to diagnoses and background variables.

The specification of measurement and structural models is guided by the psycho-social conceptual model according to the following analytic strategy::

Firstly, the one and two-factor approach (core approach) will be tested for the whole sample n(all) = 137 and for the two-group analyses n(male)=69 and n(female)=68. In searching for a multifactor solution, we assume that these factors would be correlated.

Secondly, a three-factor model is fitted where NC is differentiated into social and emotional constructs This metric model is used as a vehicle for testing direct and indirect effects of ADHD lifetime diagnose on the OC and NC social and emotional constructs.

7. New GASA measurement models

Path diagram

In latent variable modeling path diagrams are used to illustrate the item loadings in the latent variables, measurement parts, and the relationships between latent variables, the structural part.

The notation for path diagram:

1. Latent variable – circle
2. Manifest variable – square/rectangle
3. Single arrow -→ path (regression coefficient)
4. Double arrow ←→ correlation

The item variables are measured/manifest variables (represented by squares/rectangles in path diagram). In latent variable analyses the factor/latent variable is not possible to directly be

measured and is captured by indicators/items/manifest variables. The latent variable is defined as the common part of variance for the items loading in a factor, defined as the true variable (represented by circles in path diagram).

In the following presentation path diagrams concern the two factors in the GASA instrument OC and NC defined as:

- ✓ OC /Peripheral factor, items # 1, #2 and #3
- ✓ NC/Core factor, items where #4 Relapse and #6 Conflict both concern 'others' or a social component of NC and #5 Withdrawal and #7 Neglect duties are psychological indicators of emotional NC.

It should be noted that the estimate of the path/correlation coefficient between latent variables in this paper takes measurement errors into account and mirror the correlation between gaming behavior variables that are not directly measurable, such as OC and NC. The technical term is that the correlation is disattenuated. Such error-corrected correlation estimates generally is higher than when based on manifest measured variables. For this sample a factor score based correlation between f-score OC variable (item 1, 2 and 3) and f-score NC (item 4,5,6 and 7) is 0.765 computed in SPSS compared with the model-based correlation of 0.91 in Mplus (see Figure S3).

A stepwise strategy for the psychometric analyses of GASA instrument

In the psychometric analyses of GASA firstly the one-factor model with the core items is fitted (Model 1.1) followed by a two-group gender difference model (Model 1.1g). Next follow models for all 7 items in a one factor Model 1.2 and 1.2g. The two-factor model (core approach) is then fitted to the full sample (Model 2.1) and followed by two-group by gender modelbuilding (Model 2.1g, 2.1gconfigural and 2.1gmetric). Then proceeding to the three-factor model where NC is differentiated into the social and emotional parts (Model 3.1). This model has acceptable fit to the full sample n=137 while the two-group by gender measurement modelling showed that there are significant differences between male and female related to measurement and/or structural parts of the model. Model fitting results are reported in Table S2. Mplus input and output (fit statistics and extract of standardized solution) reported in Appendix.

Table S2(Table 3 in article). Goodness of fit Indexes for the one-, two- and three-factor solutions of GASA. Whole sample all n=137 and multiple-group by gender, n(female)=68, n(male)=69

Model #	Model Description	CFI	RMSEA
1.1	GASA CFA 1 core items NC all	0.994	0.051
1.1g	GASA MGCFA 1 core items NC by gender configural	0.954	0.077
1.2	GASA CFA 1 OC and NC all	0.960	0.077
1.2g	GASA MGCFA 1 OC and NC by gender configural	0.886	0.095
2.1	GASA CFA 2 all	0.973	0.065
2.1g	GASA MGCFA 2 by gender configural	0.933	0.077
2.1gc	GASA CFA 2 by gender configural with correlation errors between item 5 and 7	0.971	0.059
2.1gm	GASA MGCFA 2 by gender metric, model 2.1gc with eq constraints	0.935	0.079
3.1	GASA_CFA_3 all	0.974	0.069
3.1gm	GASA MGCFA 3 by gender metric eq constraints	0.959	0.069
3.2g.dia	GASA MCCFA 3 by gender model 3.1gm with covariate diagnose ADHD lifetime	0.954	0.067

Abbreviations: CFI, comparative fit index; RMSEA, root mean square error of approximation; GASA, game addiction scale for adolescents; CFA, confirmatory factor analyses; MGCFA, multiple-group confirmatory factor analysis; OC, over consumption; NC, negative consequences; ADHD, attention deficit hyperactivity disorder.

One factor models

Model 1.1 GASA CFA 1 core items NC all. Test of the one-factor CFA model, core factor n=137

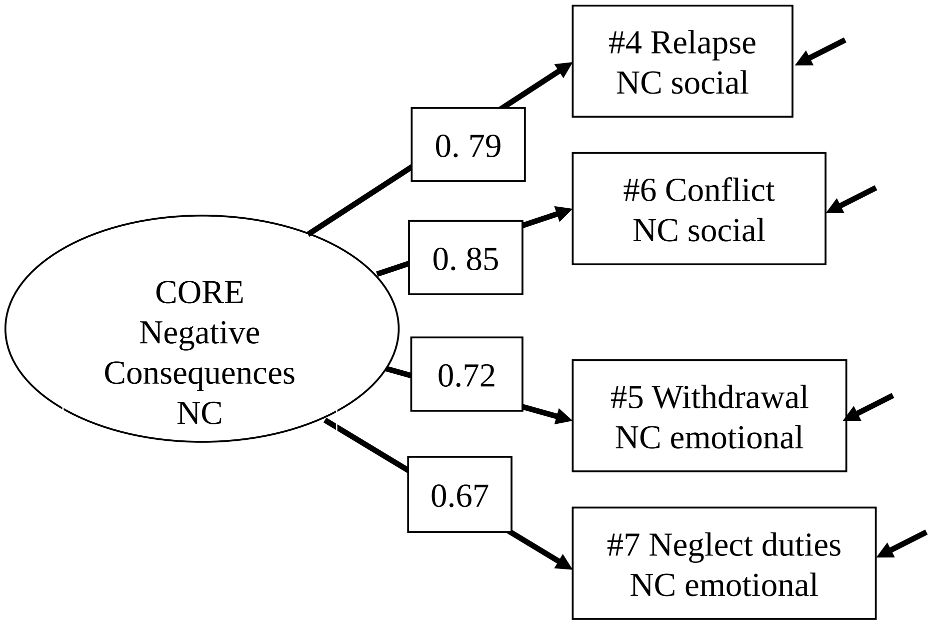

Figure S1. Model 1.1 GASA CFA one factor model of core items – problematic use of games with negative consequences.

Model 1.2 GASA CFA 1 7 items all. Test the one-factor CFA model, peripheral and core items n=137

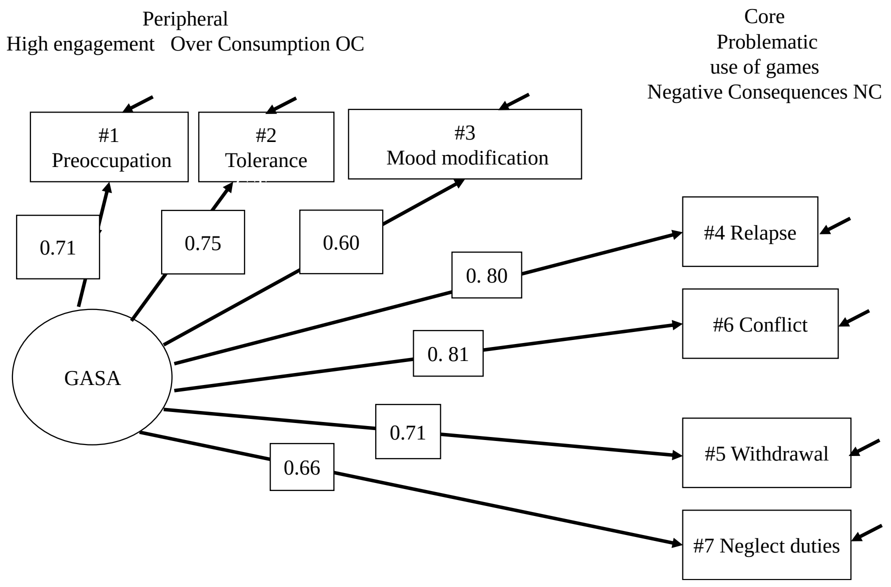

Figure S2. Model 1.2 GASA CFA one factor model with 7 items Peripheral and Core.

Two factor models

Model 2.1 GASA_CFA_2 all. Test of the two-factor CFA model, peripheral-core approach proposed by Brunborg et al (2015). The content of the peripheral items cover high engagement, Over Consumption (OC) and for the core items problematic use of games which mirror Negative Consequences (NC). The model specification is either to see over consumption as an explanatory variable for problematic use of games rather than a peripheral component or as in Figure S3 treat the two factors as correlated.

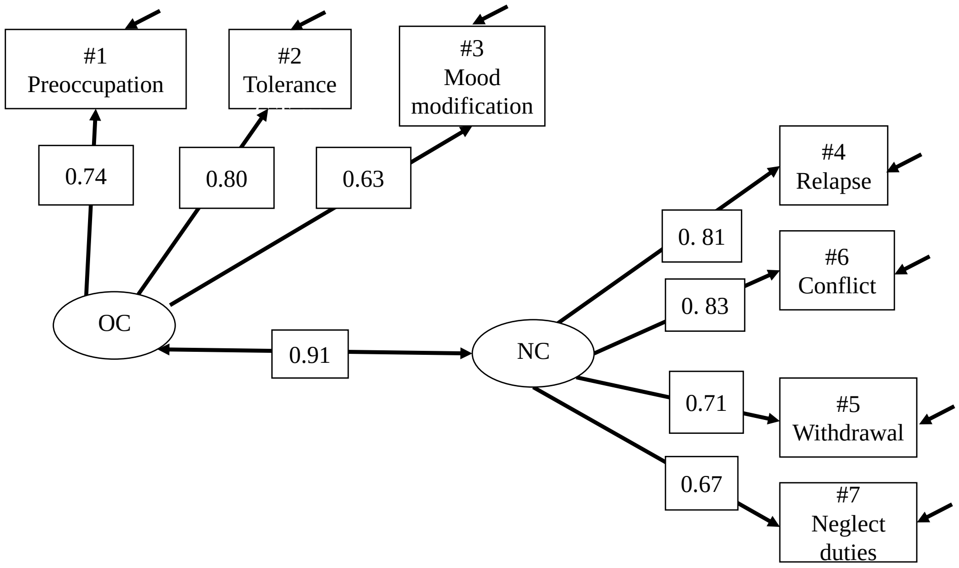

Figure S3 (Figure 1 in article). Model 2.1 – GASA CFA 2 all. Two-factor Core approach model, OC/Peripheral and NC/ Core.
Note. OC, over consumption; NC, negative consequences.

In the measurement Model 2.1 the estimate of the correlation between f(OC) and f(NC) is high, 0.91, pointing at that overconsumption is the main cause of the gaming activities that lead to negative consequences and social and emotional problems and harm.

The GASA two-factor analyses of core and peripheral items (GASA CFA 2) has an acceptable fit (CFI=0.973; RMSEA = 0.065) which confirm that core approach shows a valid factor structure for the total sample n=137 (see Figure S3 and Table S2).

Two group gender CFA models

GASA CFA models by gender.

Model 1.1g GASA CFA 1 core items NC by gender eq constraints within NC components n(female)=68, n(male)=69, RMSEA 0.077 and CFI 0.954.

Model 1.2g GASA CFA 1 GASA 7 items by gender eq constraints within NC components, RMSEA 0.095 and CFI 0.886 signing bad fit

Model 2.1g GASA_CFA_2 by gender define a two-group GASA model by gender for the two-factor model (core approach model), see Figure S4. The fit is improved when correlated errors between item 5 and 7 configural are inserted, the relationship between Withdrawal and Neglect duties items.

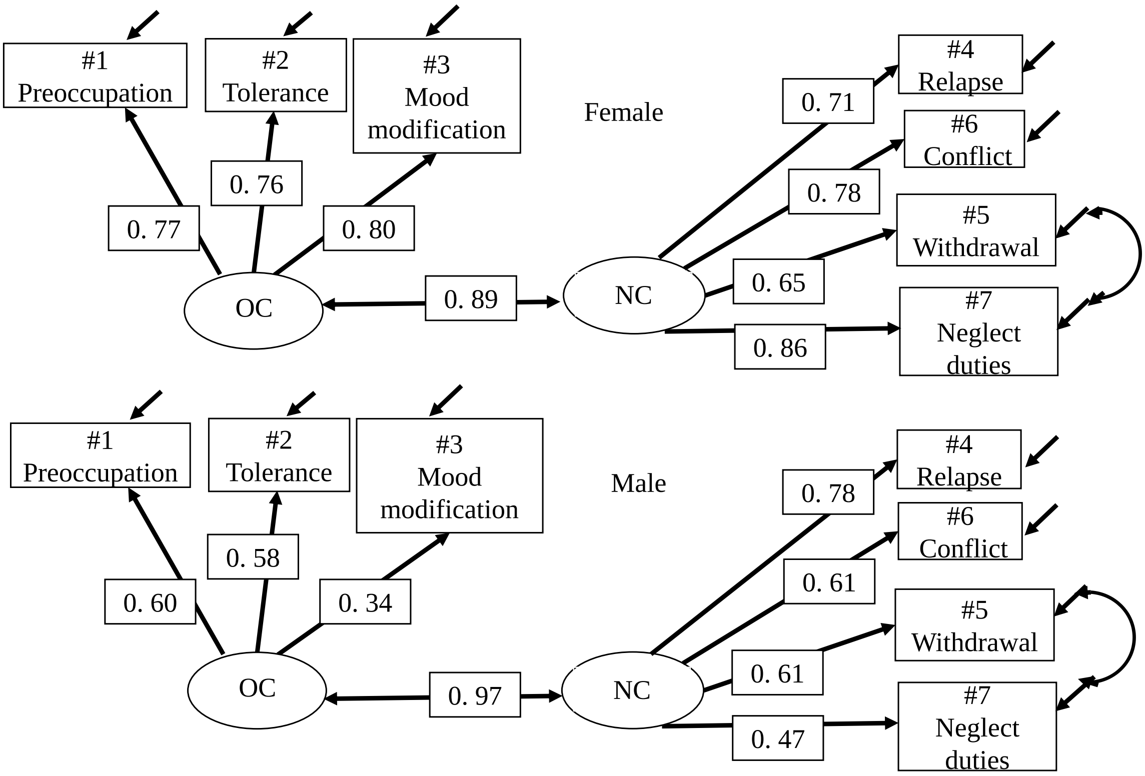

Figure S4 (Figure 2 in article). Model 2.1gc – GASA MGCFA 2 by gender configural. Two-group two-factor Core approach model, OC/Peripheral and NC/Core. Correlated errors between NC item #5 Withdrawal and #7 Neglect Duties.

Note. OC, over consumption; NC, negative consequences.

When inserting in Model 2.1g the correlation between error terms for item #5 and #7, the NC emotional items, the goodness of fit is improved, CFI=0.971 and RMSEA = 0.059 for this Model 2. gc (see Figure S4). The error correlation is significant for male (p-value = 0.066) but not for female (p-value 0.728). Another gender difference is that the correlation between OC and NC latent variables is 0.89 for female and higher 0.97 for male, that means overconsumption explains to a big extent the variation in negative consequences especially for male (see Figure S4).

The Model 2.1gc configural model is characterized by no restrictions across groups. The significant error correlation is an indication that the NC factor could be differentiated into NC social (item 4 and 6) and NC emotional (Item 5 and 7). This three-factor model is presented below.

Model 2.1gm GASA MGCFA 2 by gender metric, defined as model 2.1gc with eq constraints within NC components, resulted in RMSEA 0.079 and CFI 0.935 not acceptable fit for the two-factor metric model.

Three factor models

Model 3.1 GASA_CFA_3 all three-factor model – Over Consumption and Negative Consequences divided into NC social and NC emotional.

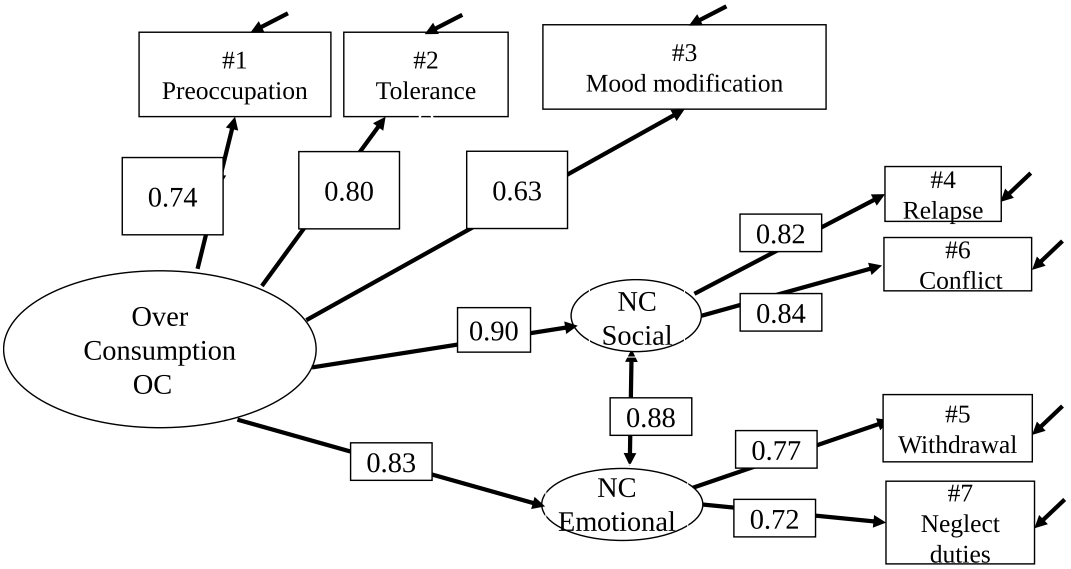

Figure S5. Model 3.1 GASA CFA three factor model all – Over Consumption and Negative Consequences divided into NC social and NC emotional.

Model 3.1gm GASA MGCFA 3 metric define a two-group GASA model by gender for a three-factor model, core items are separated into NC Social and Emotional with equality constraints for the measurement parts of the model. Another feature of this most advanced GASA measurement model is that OC is specified as an explanatory variable of the NC factors. The negative consequences are outcome variables and overconsumption representing a mediating variable as in the psycho-social conceptual model in Diagram S1.

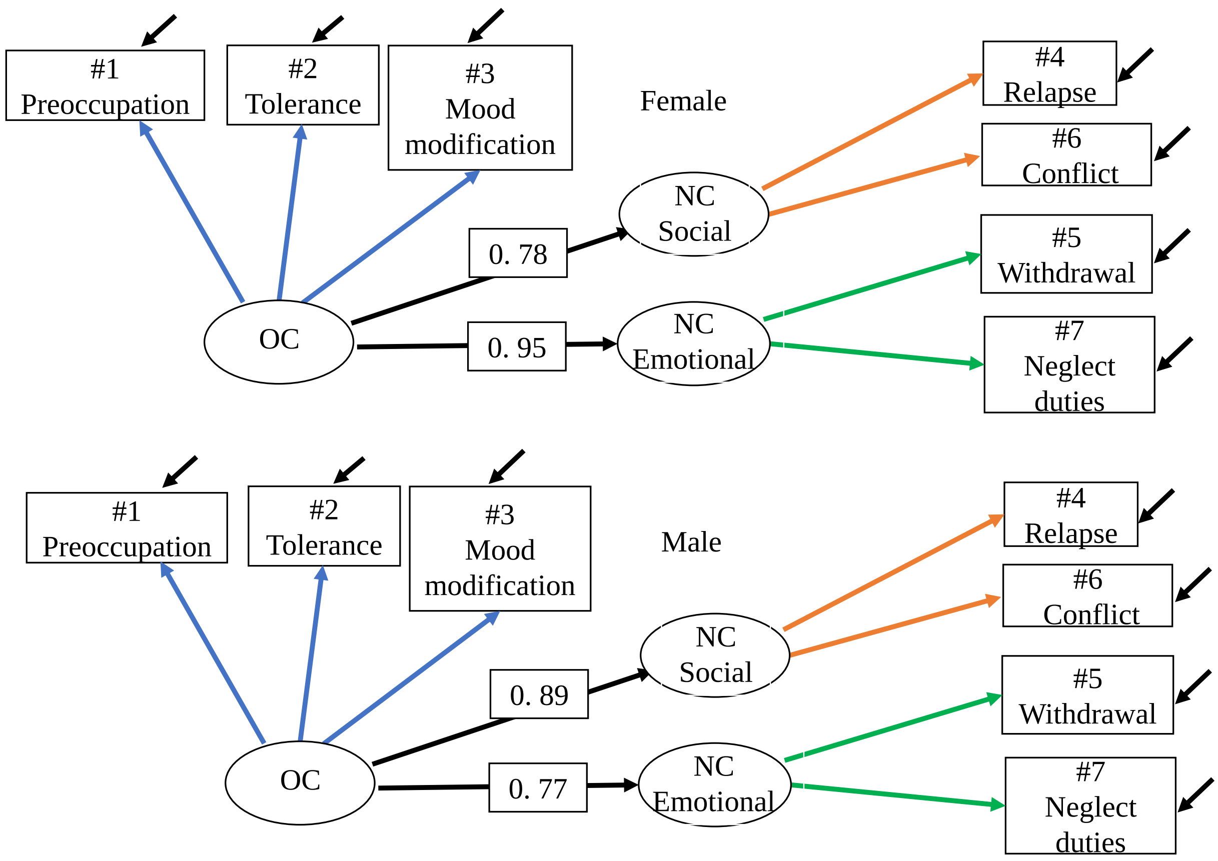

Figure S6 (Figure 3 in article). Model 3.1gm – GASA MGCFA 3 by gender, metric. Two-group three-factor model by gender with core items divided into NC social and emotional with equality constraints across gender groups for corresponding measurement models.

Note. Residual correlations NC Social with NC Emotional (not represented in the path diagram) for male is 0.40 and for female 0.87. OC, over consumption; NC, negative consequences.

The path coefficient OC -> NC equal 0.91 in the overall modell 2 shows an extraordinarily strong relationship between OC and NC see Figure S3. When this link between OC and NC in model 3 is differentiated into a Social and Emotional path the result show that for male the strongest relationship is OC-> NC Social .89 and for female OC-> NC Emotional 0.95.

These results imply that the psychometric modelbuilding could identify a three factor GASA measurement model tested for overall goodness of fit and for metric invariance for gender.

This most differentiated GASA measurement model is raising questions how the GASA instrument could be further developed to capture both social and emotional components. In general, latent variable modeling demand three reliable items to define a factor well (Price, 2017). With only two items for each factor in this self-test the social and emotional dimensions should preferably be strengthen by some supplementary items. In the 15-item adult gambling GamTest the emotional factor, measured with 5 items, was shown to be the dominant part of the general factor present in every answer applying a bifactor approach (Jonsson et al 2017).

Furthermore, in the remarkably high estimated correlation (OC, NC) = 0.91 the OC and NC true correlation is concatenated with the general factor that is captured by the GASA diagnostic instrument as a whole, a hidden factor part of every answer to the 7 questions. In self testing with questionnaires the overall circumstances in the testing situation influencing all the answers to the questions will constitute such a problematic method factor not possible to identify with only 7 items in the test and further investigation of the mode of data collection to secure a reliable communication with the patients. For example, youth motivation and reading comprehension, and answer format are important aspects to consider improving the validity of GASA.

8. Two applications.

GASA 3 factor model with covariate ADHD diagnose.

The model GASA CFA 3 in Figure S6 will be further analyzed in a SEM path model to illustrate how all three dimensions OC, NC social and emotional can be outcome variables for testing the relationship between ADHD diagnosis and Gambling behavior and Harm. In Table S3 and Figure S7 the estimated structural path coefficients are reported for GASA_CFA_3 by gender model 3.1g with covariate diagnose ADHD lifetime (Model 3.2gdia).

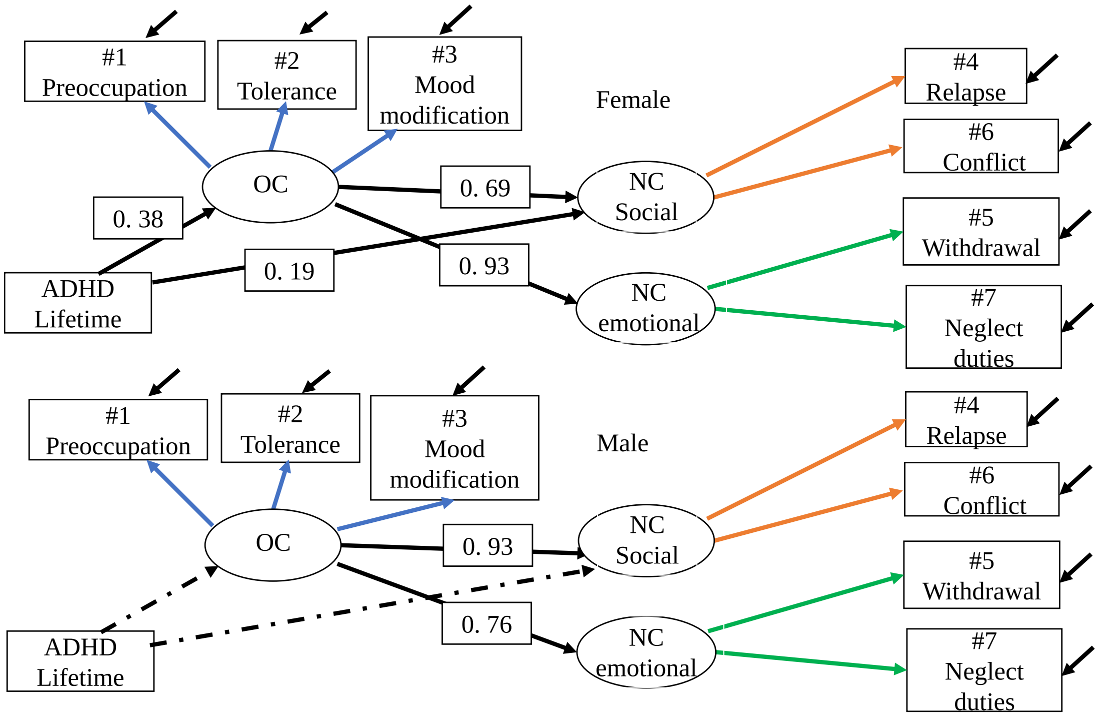

Figure S7 (Figure 4 in article). Model 3.2g.dia – GASA MGCFA 3 by gender, metric with covariate. The two-group three-factor model with equality constraints across gender for corresponding measurement models and with covariate ADHD lifetime.

Note. Dotted line is non-significant path. OC, over consumption; NC, negative consequences; ADHD, attention deficit hyperactivity disorder.

Table S3. Estimated path coefficients with test results for Model 3.1gdia GASA CFA three factor metric model by gender with equality constraints for corresponding measurement models and covariate ADHD lifetime

Group ID	Path	Estimate	S.E.	t-value	Two-Tailed p-value
Female	OC on				
	ADHD ever	0.382	0.125	3.052	0.002
	NCsocial on				
	ADHD ever	0.191	0.103	1.858	0.063
	OC	0.689	0.093	7.378	0.000
	NCmental on				
	OC	0.933	0.113	8.237	0.000
Male	OC on				
	ADHD ever	-0.144	0.150	-0.956	0.339
	NCsocial on				
	ADHD ever	0.191	0.119	1.600	0.110
	OC	0.929	0.088	10.614	0.000
	NCmental on				
	OC	0.756	0.136	5.558	0.000

Group difference testing with GASA factor score aligned variables.

Factor scores and means optimized for measurement non-invariance across gender are computed with the alignment procedure in Mplus. This solution is based on a one-factor configural model fitted to OC items and NC items separately. Factor scores and means in an alignment optimization metric are saved in Mplus for further post processing in SPSS, in Table S4 the new variables are defined (Muthén and Asparouhov 2018; Muthén and Muthén 1998-2020; Munck et al 2018; Rudnev, 2020).

Table S4. New GASA Factor Score Aligned Variables.

Variable Description	Variable Label	#1	#2	#3	#4	#5	#6	#7
Over Consumption/ Peripheral	FOC_a	x	x	x				
Negative Consequences/Core	FNC_a				x	x	x	x

Note. Model 1.1g configural model for NC/core and aligned factor score Mplus input and output see Appendix.

To achieve a common metric for table and diagram presentations the FOC_a and FNC_a variables are transformed into a common standardized T score scale for the CAP sample with mean 100 and sd 50;

TFOC Over Consumption is defined as T-score for FOC_a and

TFNC Negative Consequences is defined as T-score for FNC_a.

These new T-scores measuring severity in OC and NC in a common metric optimized for valid gender differences the CAP sample. are summarizing the mean profiles by Gender and Age groups in Figure S8 and by ADHD Lifetime diagnosis in Figure S9.

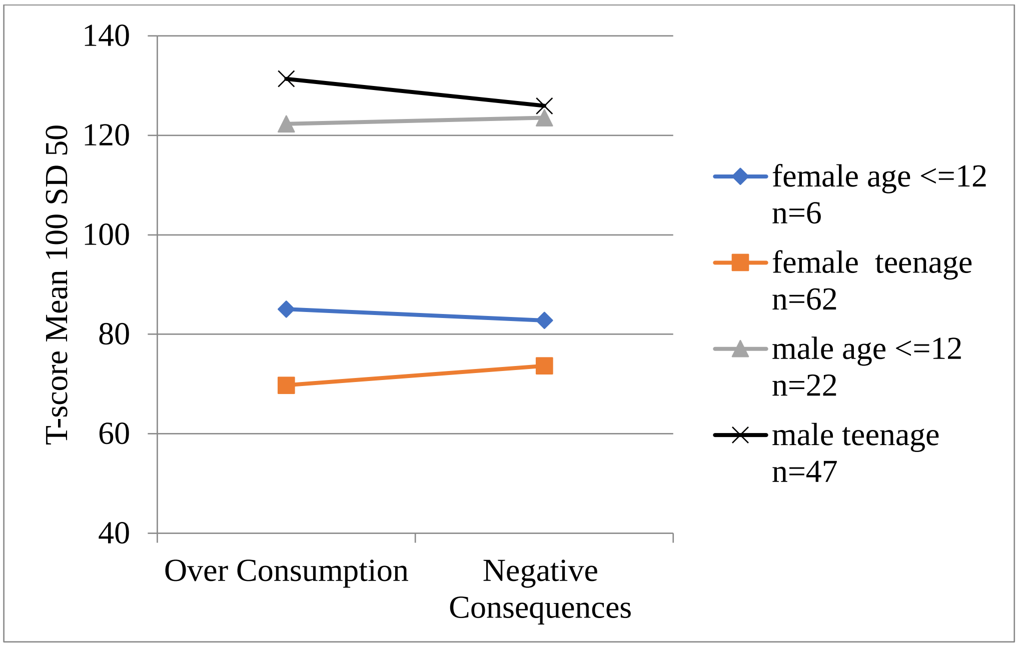

Figure S8 (Figure 5 in article). Over consumption and negative consequences mean profiles for gender by age groups. Scale is aligned factor T-scores.
Note. Scale is aligned factor T-scores. Mean 100 and SD 50 for the CAP sample. Data see Table S6.

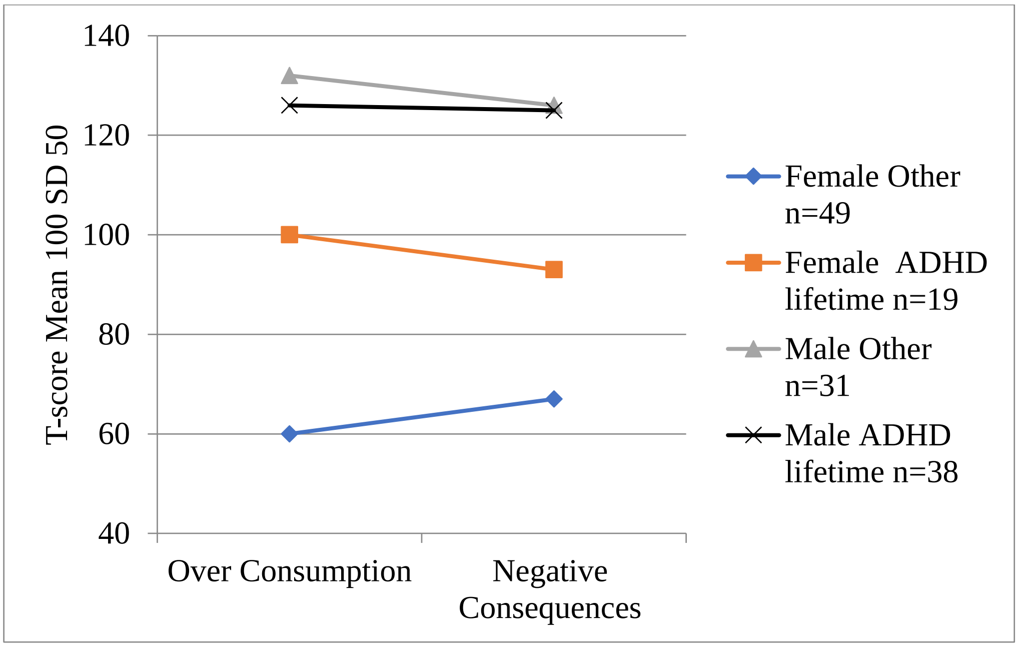

Figure S9 (Figure 6 in article). Over consumption and negative consequences mean profiles for gender by ADHD lifetime diagnosis groups.
Note. Scale is aligned factor T-scores. Mean 100 and SD 50 for the CAP sample. Data see Table S6.

ANOVA testing by gender for groupings according to Age and ADHD lifetime diagnosis are reported with SPSS procedure 'Non-parametric test for Independent-Samples Mann-Whitney U Test' not requiring the normality assumption.

Our results show a big gaming severity gap between male and female, with male gaming much more intensively than female and with more severe consequences. ANOVA F-test for OC and for NC are highly significant with p<0.00 (OC F(1,135)=66.9 p<0.000; NC F(1,135)=47.2 p<0.000).

Both for females and males there is a small difference between the child and teenage groups concerning their gaming severity in over consumption and negative consequences, see Figure S8, test results in Table S5, p-values ranging from 0.24 up to 0.88.

There is an interaction effect between gender and ADHD lifetime diagnosis illustrated in Figure S9, female shows a highly significant difference between ADHD lifetime and Other diagnoses both for Over Consumption and for Negative Consequences (p-value for OC 0.01 and for NC 0.03) while male's mean profiles are very close and non-significant but at a much higher level compared with the females (ANOVA tests reported in Table S5 and descriptive statistics in Table S6).

Table S5 Separate for female and male, one way ANOVA test for mean aligned factor score gap between diagnose ADHD lifetime or not for Core/OC and Peripheral/NC respectively.

Gender	Alinged Factor T-score by ADHD lifetime/ Age groups	Independent-Samples Mann-Whitney U Test Summary				
		Total N	Mann-Whitney U	Standard Error	Standardized Test Statistic	Asymptotic Sig.(2-sided test)
Female	OC by Age	68	179	44.73	-0.16	0.88
	NC by Age	68	162	41.27	-0.58	0.56
Male	OC by Age	69	608	77.50	1.17	0.24
	NC by Age	69	519	77.55	0.03	0.79
Female	OC by ADHD_lifetime	68	644	70.76	2.52	0.01
	NC by ADHD_lifetime	68	612	65.29	2.24	0.03
Male	OC by ADHD_lifetime	69	529	82.72	-0.72	0.47
	NC by ADHD_lifetime	69	582	82.78	-0.09	0.93

Abbreviations: OC, over consumption; NC, negative consequences; ADHD, attention deficit hyperactivity disorder.

Note 1: ANOVA test is SPSS procedure Non-parametric test for Independent-Samples Mann-Whitney U Test. Significance level is 0.05

Note 2: TF-Scale for aligned factor score is T-score with mean 100 and SD 50.

Table S6 Descriptive Statistics for aligned factor T-score by Age groups and ADHD lifetime or not for Core/OC and Peripheral/NC.

Group	Descriptive Statistics	Aligned Factor T-score for OC and NC					
		Female		Male		Total	
		TFOC	TFNC	TFOC	TFNC	TFOC	TFNC
Age 8-12 years	Mean	85	83	122	124	114	115
	Std. Deviation	75.90	45.48	25.21	44.63	42.47	47.14
	n	6	6	22	22	28	28
Age 13-18 years	Mean	70	74	131	126	96	96
	Std. Deviation	46.56	36.36	33.11	50.77	51.29	50.21
	n	62	62	47	47	109	109
ADHD lifetime	Mean	100	93	126	125	117	114
	Std. Deviation	63.74	47.71	31.05	47.89	45.77	49.67
	n	19	19	38	38	80	80
Other diagnoses	Mean	60	67	132	126	88	90
	Std. Deviation	37.53	29.23	30.90	50.20	49.61	48.01
	n	49	49	31	31	57	57
Total	Mean	71	74	128	125	100	100
	Std. Deviation	49.23	36.94	30.91	48.58	50.00	50.00
	n	68	68	69	69	137	137

Abbreviations: OC, over consumption; NC, negative consequences; ADHD, attention deficit hyperactivity disorder.

Note: TF-Scale for aligned factor score is T-score. Mean 100 and SD 50 for the CAP sample. Gaming habits among youth

9. Epilog citation from Rupp et al. 2010: *Diagnostic Measurement – Theory, Methods, and Applications*

Diagnostic classification models (DCMs) have evolved into powerful tools over the last 20 years, particularly in the areas of educational and psychological measurement (Rupp et al. 2010). These models are ideally suited to disentangle the multidimensional content of many assessment tools to provide unambiguous information about respondents that can guide decisions and help specialists make accurate diagnoses about them. In this response-contingent clinical assessment of GASA instrument, an appropriate DCM is an essential tool to accurately classify and ultimately diagnose precisely where and in what way a respondent is deficient. not having enough quality. The precision and accuracy of empirically grounded decisions with modern DCMs will become the delight of intervention-minded researchers spanning educational, clinical, and personnel assessment.

10. References

Agresti, A. and Finlay, B.(2009). *Statistical Methods for the Social Sciences*. Fourth Edition, Pearson Prentice Hall.

American Psychiatric Association. Diagnostic and statistical manual of mental disorders. 5th ed. Washington (DC): American Psychiatric Association, 2013.

André, F., Håkansson, A., Johansson, B.A., Claesdotter-Knutsson, E.(2021). The Prevalence of Gaming and Gambling in a Child and Adolescent Psychiatry Unit, submitted.

André, F. and Munck, I. (2021) Supplement: The Prevalence of Gaming and Gambling in a Child and Adolescent Psychiatry unit - Data preparation, statistical analyses and supplementary tables. Online supplement to André et al 2021.

Asparouhov, T. and Muthén, B. (2014). Multiple-group Factor Analysis Alignment. *Structural Equation Modeling.* 21: 495-508. doi: 10.1080/10705511.2014.919210.

Blaszczynski A & Nower L (2002). A pathways model of problem and pathological gambling. Addiction, 97, 487-499.

Brunborg GS, Mentzoni RA, Melkevik OR, et al (2013). Gaming Addiction, Gaming Engagement, and Psychological Health Complaints Among Norwegian Adolescents. *Media Psychol.* 16(1):115–28.

Brunborg GS, Hanss D, Mentzoni RA, Pallesen S.(2015). Core and peripheral criteria of video game addiction in the game addiction scale for adolescents. *Cyberpsychol Behav Soc Netw.* 18(5):280–5.

Jonsson, J. Munck, I. Volberg, R. & Carbring, P. (2017). GamTest: Psychometric Evaluation and the Role of Emotions in an Online Self-Test for Gambling Behavior. *Journal of Gambling Studies*, 33(2), 505-523. DOI:10.1007/s10899-017- 9676-4.

Jonsson, J. (2019). *Preventing problem gambling: Focus on overconsumption.* PhD Dissertation. Stockholm University. Stockholm. URN: urn:nbn:se:su:diva-172446.

Lemmens SJ, Valkenburg MP, Peter J.(2009).Development and validation of a game addiction scale for adolescents. *Media Psychol.*12:77–95.

Lemmens JS, Valkenburg PM, Gentile DA. (2015). The Internet Gaming Disorder Scale. *Psychol Assess.*;27:567–82.

MacCallum, R. C., Browne, M. W., & Sugawara, H. M. (1996). Power analysis and determination of sample size for covariance structure modeling. Psychological Methods, 1(2), 130–149. doi:10.1037/1082- 989X.1.2.130.

Munck, I., Barber, C., & Torney-Purta, J. (2018). Measurement invariance in comparing attitudes toward immigrants among youth across Europe in 1999 and 2009. Sociological Methods & Research, 47:4 687-728. DOI: 10.1177/0049124117729691

Muthén, B. and Asparouhov, T. (2018). Recent methods for the study of measurement invariance with many groups: Alignment and random effects. Sociological Methods & Research, 47:4 637-664. DOI: 10.1177/0049124117701488.

Muthén, L.K. and Muthén, B.O. (1998-2020). *Mplus User's Guide.* Eighth Edition. Los Angeles, CA: Muthén & Muthén. www.statmodel.com.

Muthén, B., Muthén , L.K., & Asparouhov, T. (2016). Regression and Mediation Analysis using Mplus. Los Angeles, CA: Muthén & Muthén. www.statmodel.com.

Price, L.R. (2017). *Psychometric Methods – Theory into Practice.* New York: The Guilford Press. LCCN 2016013346 | ISBN 9781462524778

Rudnev, M.(2020). *Alignment method for measurement invariance: Tutorial.* https://maksimrudnev.com/category/tutorial

Rupp, A.A, Templin, J., Henson, R.A. (2010). *Diagnostic Measurement – Theory, Methods, and Applications.* New York: The Guilford Press. ISBN 978-1-60623-528-7

Raykov, T., Marcoulides, G. A., & Tong, B. (2016). Do two or more multicomponent instruments measure the same construct? Testing construct congruence using latent variable modeling. Educational and Psychological Measurement. doi:10.1177/0013164416629714.

Appendix: GASA Psychometric analyses - Mplus Input and Output.

Model 1.1 GASA CFA 1 Core NC items All

```
TITLE:  GASA_CFA_1 mlr paper II NC
 DATA:  FILE IS GASA_paperII.dat;
 VARIABLE:
        NAMES ARE ID   Gender ADHD_lifetime Depression_lifetime
              ASD_lifetime  Anxiety_lifetime Other_lifetime Teenage
              GASA1r GASA2r GASA3r
             GASA4r GASA5r GASA6r GASA7r;
        USEVARIABLES ARE
             GASA4r  GASA5r GASA6r GASA7r;
  ANALYSIS: ESTIMATOR = MLR;
  MODEL:
        f2 BY GASA4r GASA5r GASA6r GASA7r;
  OUTPUT: SAMPSTAT  STANDARDIZED MODINDICES;
```

SUMMARY OF ANALYSIS

```
Chi-Square Test of Model Fit
      Value                          2.715
      Degrees of Freedom                 2
      P-Value                       0.2574
      Scaling Correction Factor     1.9813
      for MLR

RMSEA (Root Mean Square Error Of Approximation)
      Estimate                       0.051
      90 Percent C.I.                0.000  0.185
      Probability RMSEA <= .05              0.370

CFI/TLI

      CFI                            0.994
```

```
        TLI                            0.983
STDYX Standardization

                                           Two-Tailed
                    Estimate       S.E.  Est./S.E.    P-Value

 F2       BY
    GASA4R             0.793      0.051     15.683      0.000
    GASA5R             0.716      0.067     10.657      0.000
    GASA6R             0.847      0.042     20.192      0.000
    GASA7R             0.666      0.070      9.496      0.000
```

---------------------------------End of Model 1.1 --

Model 1.1.g GASA CFA 1 Core NC items by Gender

```
INPUT INSTRUCTIONS
  TITLE:  GASA_CFA_1 Gender MLR core NC with eq constraints within NC components
       DATA:  FILE IS GASA_paperII.dat;
       VARIABLE:
           NAMES ARE ID Gender ADHD_lifetime Depression_lifetime
                 ASD_lifetime  Anxiety_lifetime Other_lifetime Teenage
                 GASA1r GASA2r GASA3r
                GASA4r GASA5r GASA6r GASA7r;
           USEVARIABLES ARE Gender
                GASA4r GASA5r GASA6r GASA7r;
                GROUPING IS Gender (0=female 1=male);
     ANALYSIS: ESTIMATOR = MLR;
     MODEL: f2 BY  GASA4r(1)
                GASA5r(2)
                GASA6r(1)
                GASA7r(2);
     OUTPUT: SAMPSTAT TECH4 STANDARDIZED MODINDICES;
```

SUMMARY OF ANALYSIS

```
Chi-Square Test of Model Fit
        Value                          15.461
        Degrees of Freedom                 11
        P-Value                         0.1624
        Scaling Correction Factor       1.6800
          for MLR
RMSEA (Root Mean Square Error Of Approximation)
        Estimate                        0.077
        90 Percent C.I.                   0.000  0.159
        Probability RMSEA <= .05          0.280


CFI/TLI
        CFI                            0.954
        TLI                            0.949
```

STANDARDIZED MODEL RESULTS

STDYX Standardization

	Estimate	S.E.	Est./S.E.	Two-Tailed P-Value
**Group FEMALE**				
F2 BY				
GASA4R	0.806	0.082	9.824	0.000
GASA5R	0.692	0.061	11.409	0.000
GASA6R	0.825	0.068	12.195	0.000
GASA7R	0.633	0.082	7.763	0.000
Means				
F2	0.000	0.000	999.000	999.000
Intercepts				
GASA4R	1.895	0.142	13.330	0.000
GASA5R	2.562	0.218	11.778	0.000
GASA6R	1.941	0.136	14.255	0.000
GASA7R	2.597	0.185	14.036	0.000
Variances				
F2	1.000	0.000	999.000	999.000
Residual Variances				
GASA4R	0.350	0.132	2.640	0.008
GASA5R	0.522	0.084	6.219	0.000
GASA6R	0.319	0.112	2.853	0.004
GASA7R	0.599	0.103	5.797	0.000
**Group MALE**				
F2 BY				
GASA4R	0.763	0.056	13.600	0.000
GASA5R	0.605	0.075	8.088	0.000
GASA6R	0.758	0.064	11.863	0.000
GASA7R	0.583	0.066	8.892	0.000
Means				
F2	1.105	0.182	6.065	0.000
Intercepts				
GASA4R	1.253	0.107	11.702	0.000
GASA5R	1.567	0.107	14.615	0.000
GASA6R	1.246	0.089	13.964	0.000
GASA7R	1.669	0.115	14.521	0.000
Variances				
F2	1.000	0.000	999.000	999.000
Residual Variances				
GASA4R	0.418	0.086	4.877	0.000
GASA5R	0.633	0.091	6.989	0.000

```
   GASA6R          0.425      0.097      4.383      0.000
   GASA7R          0.661      0.076      8.652      0.000
```
---------------------End of Model 1.1.g---

GASA Alignment procedure for Core NC items by Gender

```
  TITLE:  GASA_CFA_1_NC mlr BY GENDER paper II
        alignment fixed
  DATA:  FILE IS GASA_paperII.dat;
  VARIABLE:
         IDVARIABLE = ID;
         NAMES ARE ID    Gender ADHD_lifetime Depression_lifetime
                 ASD_lifetime  Anxiety_lifetime Other_lifetime Teenage
                 GASA1r GASA2r GASA3r
                GASA4r GASA5r GASA6r GASA7r;
          USEVARIABLES ARE  Gender
               GASA4r GASA5r GASA6r  GASA7r;
          CLASSES = c(2);
          KNOWNCLASS = c(Gender = 0 1);
ANALYSIS:
          TYPE = MIXTURE;
          ESTIMATOR = MLR;
          ALIGNMENT = FIXED (0);
          ASTARTS = 100;
MODEL:
        %OVERALL%
        f1 BY GASA4r GASA5r GASA6r GASA7r;

OUTPUT: SAMPSTAT TECH8 ALIGN  ;
SAVEDATA: SAVE =    FSCORES;
        FILE IS GASA_ALIGN_CFA1NC;
```

Cont. in ‘Appendix to Supplement Mplus Input Output_210520.doc’ available on request Ingrid.munck@med.lu.se.
